# Genealogical lineage sorting leads to significant, but incorrect Bayesian multilocus inference of population structure

**DOI:** 10.1111/j.1365-294X.2010.04990.x

**Published:** 2011-03

**Authors:** PABLO OROZCO-terWENGEL, JUKKA CORANDER, CHRISTIAN SCHLÖTTERER

**Affiliations:** * Institut für Populationsgenetik, Vetmeduni ViennaVeterinärplatz 1, 1210 Vienna, Austria; † Department of Mathematics and Statistics, University of HelsinkiHelsinki FIN-00014, Finland

**Keywords:** confidence of inference, *Drosophila melanogaster*, microsatellites, population structure

## Abstract

Over the past decades, the use of molecular markers has revolutionized biology and led to the foundation of a new research discipline—phylogeography. Of particular interest has been the inference of population structure and biogeography. While initial studies focused on mtDNA as a molecular marker, it has become apparent that selection and genealogical lineage sorting could lead to erroneous inferences. As it is not clear to what extent these forces affect a given marker, it has become common practice to use the combined evidence from a set of molecular markers as an attempt to recover the signals that approximate the true underlying demography. Typically, the number of markers used is determined by either budget constraints or by statistical power required to recognize significant population differentiation. Using microsatellite markers from *Drosophila* and humans, we show that even large numbers of loci (>50) can frequently result in statistically well-supported, but incorrect inference of population structure using the software baps. Most importantly, genomic features, such as chromosomal location, variability of the markers, or recombination rate, cannot explain this observation. Instead, it can be attributed to sampling variation among loci with different realizations of the stochastic lineage sorting. This phenomenon is particularly pronounced for low levels of population differentiation. Our results have important implications for ongoing studies of population differentiation, as we unambiguously demonstrate that statistical significance of population structure inferred from a random set of genetic markers cannot necessarily be taken as evidence for a reliable demographic inference.

## Introduction

With the advent of PCR, the 1980s saw the dawn of the field of phylogeography, a discipline that deals with the study of processes that lead towards the observed distribution of genetic variation within and between populations or species in a geographical and temporal context. In its early stages, it focused on the distribution of mitochondrial (mtDNA) genetic variation (Avise *et al.* 1987; Avise 2001). Nonetheless, mtDNA represents a single locus, and as expected by the stochasticity of the coalescent process (Kingman 1982), its genealogy may not reflect that obtained with other independent molecular markers such as nuclear microsatellites (Avise 2001; Brito & Edwards 2009; Degnan & Rosenberg 2009; Than & Nakhleh 2009).

Inference of population structure is at the core of phylogeographic studies as it reflects divergence between populations. While the primary focus in phylogeographic studies is on population differentiation caused by genetic drift, it must be kept in mind that some genomic regions are also affected by selection. Contrasting the pattern of population structure for different genomic regions has been advocated as an approach to distinguish between neutrally evolving and selected regions in the genome (Lewontin & Krakauer 1973; Beaumont 2005; Excoffier *et al.* 2009; Turner *et al.* 2010). Furthermore, identification of the underlying population structure is also important for other research areas such as personalized medicine. Neglecting population subdivision can lead to development of drugs with undesired population-specific phenotypical responses (Wilson *et al.* 2001). Moreover, not accounting for population structure will result in a high false discovery rate in association studies (Holsinger & Weir 2009; Kang *et al.* 2010; Zhang *et al.* 2010). Hence, a reliable identification of population structure is of utmost importance as it reflects past biological processes that can explain the distribution of genetic variation.

For most species, the characterization of population structure is still limited by the availability of informative markers. Microsatellites are a very powerful tool for such studies as their high polymorphism and mutation rates allow differentiating even between recently diverged populations or species (Goldstein & Pollock 1997; Schlötterer 2001; Hofer *et al.* 2009). While microsatellites are highly abundant in most species, their isolation requires a considerable investment, thus many studies rely on only a handful of microsatellites (<50 markers) to make inferences on the evolutionary history of populations and species (Dirienzo *et al.* 1994; Beaumont *et al.* 2001; Chiari *et al.* 2006; Lukoschek *et al.* 2008).

In this study, we analyse how the number of markers and their chromosomal location affect the inference of population structure using the software baps. We demonstrate that different combinations of microsatellite markers often result in significantly different inference of population structure. Most importantly, each of the different clustering solutions found is statistically well supported with posterior probabilities larger than 0.95. The smaller the number of markers, the more pronounced this effect is. For the data set of *Drosophila melanogaster* used here, a consistent genetic mixture model was obtained only when more than 120 loci were included, i.e. in such analyses, we found that the inferred population structure was always the same. Interestingly, we do not only detect this effect in the data of *D. melanogaster* but also in a large human data set (Rosenberg 2006).

## Materials and methods

### Microsatellite design

Previous studies surveyed the genetic diversity of *Drosophila melanogaster* using multiple genetic markers scattered across its genome (Dieringer & Schlötterer 2003; Glinka *et al.* 2003; Ometto *et al.* 2005; Schlötterer *et al.* 2006; Nunes *et al.* 2008). Contrary to these studies, we inferred population structure using markers restricted to 16 different genomic regions. On average, each of these regions encompasses 83.3 (±16) kb, and the microsatellites within them are spaced by 11 kb (±2.5 kb) (Fig. S1, Supporting information). Each chromosome is represented by five such regions except the 4th chromosome that is represented by a single region. Table 1 provides detailed information about the position of the markers on each chromosome using the *D. melanogaster* genome release 5.1 as reference. Throughout the manuscript, we refer to the chromosomal regions using the nomenclature in Table 1. Using this design, it is possible to compare population structure inferred from (i) 16 different genomic regions, (ii) entire chromosomes, and (iii) the whole genome.

**Table 1:** *Drosophila melanogaster* regions’ description. Position of regions according to cytological bands and physical distance. Physical distance is measured in base pairs within each Muller element

	Region name abbreviation	Cytological position	Physical distance	Number of loci
X region 1	Xr1	2D1–2D5	1 939 294–2 007 054	8
X region 2	Xr2	4C2–4C4	4 241 724–4 345 590	8
X region 3	Xr3	8A4–8B2	8 573 624–8 675 060	12
X region 4	Xr4	11B5–11B10	12 460 031–12 533 695	12
X region 5	Xr5	14A8–14B1	15 907 856–16 003 183	8
2 region 1	2r1	21F3–21F4	1 229 519–1 279 955	8
2 region 2	2r2	27E1–27E3	7 087 509–7 161 029	8
2 region 3	2r3	33E10–33F2	12 568 931–12 658 369	8
2 region 4	2r4	46C4–46D1	5 804 822–5 891 266	8
2 region 5	2r5	57E9–57F2	17 401 824–17 481 742	8
3 region 1	3r1	62A5–62A10	1 552 864–1 662 817	8
3 region 2	3r2	65D3–65D6	6 903 595–6 971 788	8
3 region 3	3r3	70C3–70C4	13 711 295–13 794 749	8
3 region 4	3r4	89F3–90A2	13 062 204–13 140 538	8
3 reigon 5	3r5	98B2	23 528 849–23 600 270	8
4 region	4r1	102B5–102C1	366 587–467 371	9

### Microsatellite data

DNA was extracted from single females of iso-female lines collected from 21 localities around the world (569 samples; Table 2). These samples were genotyped for 137 microsatellites (Table S1, Supporting information; the genotype data set has been deposited in Dryad: http://dx.doi.org/10.5061/dryad.8038). The microsatellite primer pairs were designed based on the *D. melanogaster* genome sequence available in Flybase (http://www.flybase.org) using Primer3 (Rozen & Skaletsky 2000). Multiplex PCRs were carried out for sets of 10 microsatellite primer pairs at a time using fluorescently labelled forward primers (Hex, Tet and Fam). Each twenty microliters PCR reaction consisted of 100 ng of genomic DNA as template, 3.2 μL of Buffer B 10×, 2 μL MgCl_2_ 25 mm, 0.4 μL dNTPs (10 mm each), 0.2 μL of each primer (20 μm) and 0.4 μL of *Taq* polymerase (5 U/μL). PCR products were analysed on a MegaBACE-500 Sequencer (GE Healthcare) and scored with Genetic Profiler (v2.2, GE Healthcare).

**Table 2:** *Drosophila melanogaster* samples’ description. Country and location refer to the geographical location where the samples come from. *n* is the number of isofemale lines collected in the corresponding location. F1 and inbred indicate whether the samples used in this study are the first offspring generation of the isofemale lines collected in the wild (F1) or if they are an unknown generation, which has been subject to inbreeding (inbred)

Country	Location	Abbreviation	*n*	F1/Inbred
China	Heilongiiang	Chi	38	F1
Taiwan	Hsin Chu	Hchu	11	Inbred
Malaysia	Kuala Lumpur	KL	20	Inbred
Philippines	Cebu	CEB	20	Inbred
Poland	Katowice	Kat	40	F1
The Netherlands	Texel	Tex	40	F1
Italy	Napoli	Np	30	F1
Austria	Vienna	KaBe	38	F1
Portugal	Evora	Evo	29	F1
Germany	Neustadt	Neu	30	Inbred
USA	Penn State	Pe	15	F1
USA	New Jersey	NJ	30	F1
Belize	La Milpa	Bel	16	F1
Brazil	Campinas	Cbr	33	F1
Bolivia	Unknown	BOL	19	Inbred
Australia	Wooton	Woo	36	Inbred
Australia	Moruya	Mor	31	Inbred
Australia (Tasmania)	Cygnet	Cyg	30	Inbred
Australia (Tasmania)	Trial Bay Orchard	Tbo	26	Inbred
Zimbabwe	Sengwa	Zs	13	Inbred
Zimbabwe	Victoria Falls	Zw	24	Inbred

### Analysis

Initially we determined if our markers recovered previously reported patterns of population structure and genetic diversity in *D. melanogaster* when analysing (i) all markers simultaneously and (ii) markers separated by chromosome. We further dissected how the inferred population structure changed when the different chromosomal regions were analysed individually. For all analyses, we inferred the pattern of population structure using the group-based clustering approach implemented in baps 5.2 (Corander & Marttinen 2006). As previous work suggested small, but significant differences among cosmopolitan *D. melanogaster* samples, we performed the genetic mixture analysis at the level of populations instead of individuals. The latter would also be possible using the options available in baps software; however, the bootstrap analyses would be computationally much more time-consuming. In addition, statistical power to correctly detect the underlying population structure is increased by the conditioning on the sample groups when it is biologically feasible (Corander & Marttinen 2006). A major part of the increase in power stems from the fact that in clustering of populations, the prior probability mass is distributed over an enormously smaller set of biological hypotheses compared to the situation where sampled individuals can be freely clustered into groups. Under a typical evolutionary scenario, the larger set of hypotheses about genetic population structure defined by clustering of individuals contain a considerable fraction of population structures that are extremely implausible in the light of sampling design and auxiliary knowledge about the organism. Thus, when a uniform prior distribution over clusterings of individuals is used, the implausible hypotheses are given disproportionate amount of prior support. In contrast, when clustering of populations is adopted, most implausible hypotheses about genetic population structure underlying the samples are assigned prior probability equal to zero (Corander & Marttinen 2006). A recent enhancement of the structure software exploits similar reasoning (Hubisz *et al.* 2009). For all baps analyses, we assumed a uniform prior distribution of the number of clusters ranging from 1 to the maximum number of groups in the analysis, e.g. 21. We confirmed the results from the analyses by repeating each run three times (results not shown). We computed standard summary statistics (e.g. heterozygosity) in MSA 4.05 (Dieringer & Schlötterer 2003). For the expected heterozygosity, which is influenced by the random loss of allelic variation because of inbreeding in isofemale lines, we report the average heterozygosity calculated from 200 data sets where in each of them, one of the alleles was randomly discarded from each individual.

### Comparison of clustering solutions

Comparing the clustering solutions from different data sets (same populations but different loci) is not a straightforward task. If different clustering solutions are obtained, it is necessary to assess their statistical support and whether they significantly differ from each other. It is not possible to contrast the marginal likelihoods of clustering solutions directly as these values depend on the number of markers used and their information content (i.e. number of alleles, gene diversity). Hence, rather than comparing two clustering solutions directly, we determined their relative compatibility with respect to a set of reference loci. This approach is computationally considerably simpler than a direct comparison of concordance of the obtained clusters. Using, for instance, the adjusted Rand Index (Rand 1971) would necessitate the storage of all obtained clustering solutions for different data sets. Specifically, the following steps were performed in our procedure:

(i) baps was run to determine the best clustering solution of the data set of interest (test data set). (ii) The same number of loci as in the test data set was sampled without replacement from a larger data set that excluded the loci from the test data set (random data set). (iii) baps was run on the random data set and the marginal likelihood of the best clustering solution given the random data set was recorded (ml_random_). (iv) The marginal likelihood (ml) of the clustering solution from the test data set when applied to the random data set was determined (ml_test_) and (v) the difference between these values (ml_test_ and ml_random_) was calculated. If the test data set and the random data set result in the same clustering solution, then the difference in ml is zero (or very close to zero). Note that this procedure compares the ml of two clustering solutions with the same data set (i.e. the random data set), thus eliminating the problems mentioned earlier. (vi) Steps 2–5 were repeated 10 000 times to obtain a distribution of differences in ml-values. (vii) Test data sets were compared pairwise to each other with a two-sample non-parametric Kolmogorov–Smirnov (KS) test using the ml-difference distributions. The KS test was calculated with r 2.9.1 (R Development Core Team 2009). A significant KS test indicates that the distributions of ml-differences of two test data sets differ from each other, indicating that the clustering solutions of the two data sets are different. For the comparison among regions, the test data set consisted of one region. The random data set consisted of the remaining markers on the same chromosome or the markers in a different chromosome. For the chromosome-wise comparison, the test data set was the chromosome and the random data set consisted of all remaining markers in the data set.

### Genetic features that affect the accuracy of demographic inference

As the genomic regions exhibit different genetic features such as their sequence length, the number of genes contained or average heterozygosity, we sought to determine if such features could explain why regions differed in how well the markers recovered the correct clustering solution. For this purpose, we computed linear models using the features of interest as explanatory variables (*x*) and the marginal likelihood (from conditioning the complete data set on the clustering solution of each region) as response variable (*y*). We tested the following features as explanatory variables (calculated for each region): the length of the region, the number of genes annotated, the number of transposable elements present, the number of non-coding RNAs annotated, the presence/absence of inversions, the average heterozygosity, the average *F*_ST_, the average θ estimated from gene diversity and the stepwise mutation model, and the recombination rate (Fiston-Lavier *et al.* 2010). For the tests involving average heterozygosity and θ estimates, we repeated the analyses using the non-African populations only to avoid obscuring any potential signal in the data because of higher genetic variability of the African populations (Begun & Aquadro 1993; Caracristi & Schlötterer 2003). These analyses were performed in r v 2.9.1.

### Effect of divergence on the inference of population structure

To determine the effect of divergence on the inference of population structure, we simulated five populations that simultaneously diverged from their common ancestor (i.e. an unresolved polytomy) and presented on average the same divergence from each other as measured with pairwise *F*_ST_. We simulated four scenarios with a different degree of divergence between populations, i.e. an average pairwise *F*_ST_ of 0.01, 0.05, 0.1 or 0.15. Each simulated population was represented by 50 individuals and 1000 independent loci. The simulations were produced with ms and converted to microsatellite data with ms2ms.pl (Pidugu & Schlötterer 2006) (ms command lines are available in Data S1, Supporting information). We performed 1000 random draws of sets of *n* loci (*n*=10, 20, …, 100) from the data set of 1000 simulated loci using baps. For each data set, we counted (i) how many times the simulated population structure (five clusters) was inferred, (ii) how many different clustering solutions were found, and (iii) whether clustering solutions other than the simulated one had high statistical support (i.e. posterior probability (pp): >0.95).

### Genealogical lineage sorting

**Fig. 1:**
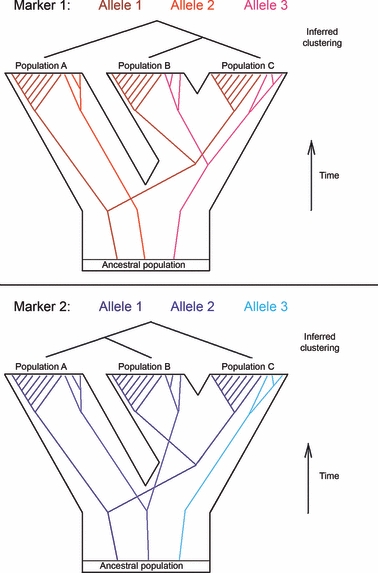
Genealogical lineage sorting. The black contour lines indicate the population history of three populations that recently diverged from a common ancestor. The two panels show the genealogy of two loci. For each locus, three alleles are shown by different colours and individuals are represented by the tips of the tree. Owing to random drift, the alleles are differently assigned to the three populations (genealogical lineage sorting). Importantly, the genealogical lineage sorting differs between the two loci, resulting in a different clustering solution. While the inferred clustering is concordant with the population history for locus 1, a different clustering is obtained for locus 2.

We considered three recently diverged populations (A, B and C) genotyped with five classes of markers. The classes were defined as follows: the first class recovers the true underlying structure of the populations with population B clustering with C and separately from A [i.e. A(BC)] (Fig. 1). The 2nd and 3rd class of markers result in clustering solutions different from the true underlying structure, i.e. population A clusters with either B or C, respectively, and separately from the third population [e.g. B(AC)]. The 4th class results in a single population cluster [i.e. (A,B,C)], and the 5th class results in each population clustering separately from the others [i.e. (A)(B)(C)]. To show the effect of genealogical lineage sorting, we sampled with replacement 1000 times a set of *n* loci from a distribution of the five marker types. We considered eight distributions of the five markers. The relative frequency of makers of class 2–5 were kept equal, while the frequency of the 1st type of marker (supporting the true clustering) was varied between 20% and 90%. For each of the random draws of *n* loci, we used a majority rule to determine which of the patterns of population structure was supported by the data set [i.e. A(BC), (AB)C, (AC)B, (ABC) or (A)(B)(C)]. We repeated this process for values of *n* in a range from 10 to 100.

### Frequency of the clustering solutions

As inference of population genetic structure is frequently based on substantially fewer markers than in this study, we aimed to determine the number of loci required to obtain the clustering solution obtained with the full data set. For this purpose, we performed 1000 random draws of a set of *n* loci from the full microsatellite data set (i.e. 137 markers). We analysed each of the random subsets of loci with baps and determined the frequency of the expected clustering solution among the 1000 random draws. This procedure was repeated for values of *n* in the range from 4 to 136. However, it should be noted that for values of *n* close to the upper limit, the random subsets will be overlapping to a large degree because of the limited number of all possible subsets of the total set of loci.

As our results based on the *D. melanogaster* microsatellites may be specific to our data set, we compared them to a neutrally simulated data set of independent loci produced with the program ms (Hudson 2002) to assert their repeatability. The coalescence simulations were performed in such way that (i) they reflected the evolutionary history of 13 *D. melanogaster* populations (two African, six European, two North-American and three Asian populations), i.e. showed a high differentiation between African and Non-African populations, a reduced genetic divergence between European and North-American populations and the reported higher divergence between the Asian populations (Schlötterer *et al.* 2006) and (ii) that summary statistics (pairwise population *F*_ST_, θ, expected heterozygosity and allelic richness) calculated from 137 randomly picked loci among the simulated ones would match those from our full microsatellite data set (ms command line available in Data S1, Supporting information). The ms outputs were converted to microsatellite data following the stepwise mutation model using the script ms2ms.pl and analysed with MSA and baps.

We repeated the analysis performed on the *D. melanogaster* data set on the curated extensive human microsatellite data set H971 (Ramachandran *et al.* 2005; Rosenberg 2006) and considering the Bantu SW and SE as different populations (Romero *et al.* 2009) (Tables S2 and S3, Supporting information).

## Results

Our analyses based on 137 polymorphic microsatellites confirm several features of the global pattern of variation in *Drosophila melanogaster* and reflects the pattern of population differentiation inferred by *F*_ST_ (Table 3). African populations harbour more genetic variation than non-African populations (Table S4, Supporting information). This trend was significant for all major chromosomes (Wilcoxon rank sum test *P-value* <0.01), which is consistent with previous reports (Begun & Aquadro 1993; Kauer *et al.* 2002). After correcting for the different effective population sizes of the X chromosome and the autosomes (Kauer *et al.* 2002), the reduction in variation was slightly more pronounced on the X chromosome than on the major autosomes. This observation is consistent with previous studies (Kauer *et al.* 2002), but we note that the difference is not statistically significant for the fourth chromosome (Wilcoxon rank sum test, *P* = 0.297).

**Table 3:** Pairwise divergence between *Drosophila melanogaster* natural populations. Above the diagonal *F*_ST_ and below the diagonal the Bonferroni corrected probabilities of significance

	Bol	CBr	Bel	NJ	Pe	Ceb	Chi	Hchu	KL	Evo	KaBe	Kat	Neu	Np	Tex	TBO	Cyg	Woo	Mor	ZS	ZW
Bol	—	0.164	0.139	0.096	0.111	0.267	0.218	0.151	0.228	0.113	0.149	0.143	0.119	0.144	0.121	0.165	0.159	0.103	0.096	0.232	0.219
CBr	0.021	—	0.115	0.075	0.079	0.195	0.148	0.094	0.158	0.087	0.101	0.102	0.080	0.093	0.093	0.140	0.132	0.085	0.082	0.203	0.193
Bel	0.021	0.021	—	0.047	0.061	0.228	0.176	0.082	0.212	0.083	0.106	0.119	0.089	0.118	0.109	0.121	0.119	0.059	0.066	0.178	0.174
NJ	0.021	0.021	0.021	—	0.003	0.168	0.122	0.046	0.147	0.024	0.046	0.046	0.022	0.041	0.038	0.066	0.060	0.029	0.015	0.176	0.164
Pe	0.021	0.021	0.021	n.s.	—	0.176	0.127	0.041	0.150	0.025	0.043	0.042	0.021	0.044	0.037	0.066	0.057	0.027	0.011	0.174	0.162
Ceb	0.021	0.021	0.021	0.021	0.021	—	0.092	0.102	0.128	0.176	0.178	0.189	0.173	0.186	0.160	0.220	0.217	0.149	0.165	0.252	0.236
Chi	0.021	0.021	0.021	0.021	0.021	0.021	—	0.053	0.096	0.123	0.125	0.133	0.116	0.126	0.106	0.167	0.163	0.105	0.117	0.246	0.234
Hchu	0.021	0.021	0.021	0.021	0.021	0.021	0.021	—	0.086	0.055	0.066	0.073	0.057	0.074	0.057	0.108	0.100	0.034	0.041	0.160	0.159
KL	0.021	0.021	0.021	0.021	0.021	0.021	0.021	0.021	—	0.144	0.156	0.160	0.143	0.146	0.131	0.206	0.203	0.132	0.140	0.255	0.240
Evo	0.021	0.021	0.021	0.021	0.021	0.021	0.021	0.021	0.021	—	0.039	0.036	0.012	0.027	0.032	0.056	0.050	0.031	0.021	0.199	0.187
KaBe	0.021	0.021	0.021	0.021	0.021	0.021	0.021	0.021	0.021	0.021	—	0.015	0.020	0.025	0.046	0.084	0.072	0.047	0.041	0.222	0.210
Kat	0.021	0.021	0.021	0.021	0.021	0.021	0.021	0.021	0.021	0.021	0.021	—	0.023	0.024	0.049	0.074	0.066	0.052	0.041	0.218	0.206
Neu	0.021	0.021	0.021	0.021	0.021	0.021	0.021	0.021	0.021	0.021	0.021	0.021	—	0.017	0.022	0.054	0.051	0.030	0.021	0.204	0.194
Np	0.021	0.021	0.021	0.021	0.021	0.021	0.021	0.021	0.021	0.021	0.021	0.021	0.021	—	0.034	0.064	0.058	0.052	0.040	0.216	0.200
Tex	0.021	0.021	0.021	0.021	0.021	0.021	0.021	0.021	0.021	0.021	0.021	0.021	0.021	0.021	—	0.078	0.068	0.043	0.034	0.209	0.200
TBO	0.021	0.021	0.021	0.021	0.021	0.021	0.021	0.021	0.021	0.021	0.021	0.021	0.021	0.021	0.021	—	0.008	0.064	0.057	0.248	0.228
Cyg	0.021	0.021	0.021	0.021	0.021	0.021	0.021	0.021	0.021	0.021	0.021	0.021	0.021	0.021	0.021	n.s.	—	0.059	0.052	0.245	0.227
Woo	0.021	0.021	0.021	0.021	0.021	0.021	0.021	0.021	0.021	0.021	0.021	0.021	0.021	0.021	0.021	0.021	0.021	—	0.018	0.178	0.172
Mor	0.021	0.021	0.021	0.021	n.s.	0.021	0.021	0.021	0.021	0.021	0.021	0.021	0.021	0.021	0.021	0.021	0.021	0.021	—	0.190	0.182
ZS	0.021	0.021	0.021	0.021	0.021	0.021	0.021	0.021	0.021	0.021	0.021	0.021	0.021	0.021	0.021	0.021	0.021	0.021	0.021	—	0.020
ZW	0.021	0.021	0.021	0.021	0.021	0.021	0.021	0.021	0.021	0.021	0.021	0.021	0.021	0.021	0.021	0.021	0.021	0.021	0.021	0.021	—

n.s., not significant.

**Fig. 2:**
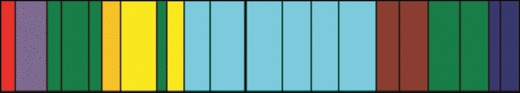
baps clustering solution for the full data set. Population order from left to right: Bolivia, Brazil, Belize, New Jersey (USA), Pennsylvannia (USA), Cebu (Philippines), China, Hsin-Chu (Taiwan), Kuala Lumpur (Malaysia), Evora (Portugal), Kahlenberg (Austria), Katowice (Poland), Neustadt (Germany), Naples (Italy), Texel (The Netherlands), Trial-Bay (Tasmania), Cygnet (Tasmania), Wooton (Australia), Moruya (Australia), Sengwa (Zimbabwe), Victoria Falls (Zimbabwe). Each population is represented by a single rectangle that is coloured according to the cluster to which the population belongs to (e.g. the two African populations belong to the same cluster). Cluster 1: Bolivia (red), Cluster 2: Brazil (purple), Cluster 3: North-America/Australia (green), Cluster 4: Cebu (light orange), Cluster 5: China/Kuala Lumpur (yellow), Cluster 6: Europe (light blue), Cluster 7: Tasmania (brown) and Cluster 8: Africa (dark blue).

Using the full data set of 137 loci, we used a model-based clustering method for multilocus data as implemented in baps (using the option of clustering of groups) and obtained eight distinct clusters (posterior probability=1) (Fig. 2). These eight clusters support the well-characterized distinction between African and non-African *D. melanogaster* (Begun & Aquadro 1993; Caracristi & Schlötterer 2003), as well as a separation of the European, North-American and Asian populations (Schlötterer *et al.* 2006; Nunes *et al.* 2008). Interestingly, the Chinese population and the Kuala Lumpur population previously reported to cluster separately from each other (Schlötterer *et al.* 2006; Nunes *et al.* 2008) belong to the same cluster in our analysis.

We repeated the population structure analysis by splitting the data according to chromosomal location. Contrary to expectations, the chromosome-based analysis yielded different clustering solutions for each chromosome with respect to the total data set (Fig. S2, Supporting information). For the X chromosome, the European population of Evora clustered with the North-American/Australian populations. The 2nd chromosome data set showed a lack of differentiation between the North-American/Australian and the European populations, and between the Asian populations. The 3rd chromosome data grouped Texel (Europe) separately of the remaining European populations; while for the 4th chromosome, the four Asian populations clustered together and the Tasmanian populations were grouped in the same cluster with the populations of Evora, Texel and the North-American/Australian populations.

An even greater diversity of different clustering solutions was obtained when we used sets of eight or 12 microsatellites separated by no more than 14 kb (Fig. S3, Supporting information). The number of clusters varied between a minimum of five (Xr3) and a maximum of eight (multiple regions on different chromosomes), and only Xr2 resulted in the same clustering solution as the full data set. Interestingly, the statistical support (i.e. posterior probability) was high (≥0.90) for all but two of the 16 regions. Lower support was obtained for region 3r4 (pp = 0.65) and 2r4 (pp=0.85).

### Significant heterogeneity in clustering among genomic regions

Our analyses indicated that even though all genomic regions resulted in different clustering solutions, most of them were statistically well supported as reflected by their high posterior probabilities. However, it is not clear from this analysis whether these optimal clustering solutions are significantly different from each other. Based on comparisons using a common reference data set (see Materials and methods), we found that the clustering solutions of different genomic regions significantly differed from each other (Kolmogorov–Smirnov test, *P*<0.0004 after Bonferroni correction; Data S1, Supporting information). A chromosome-wise analysis also resulted in all pairwise comparisons significant (*P*<0.008 after Bonferroni correction). Visual inspection of the distributions of ml-differences for each region comparison against random data sets of the three major chromosomes (Fig. S4, Supporting information) confirmed that the regions differ in their ml-differences. While some regions have a narrow distribution centred on small ml-differences, others have a broad distribution with large ml-differences.

### Predictive power of different marker sets

**Fig. 3:**
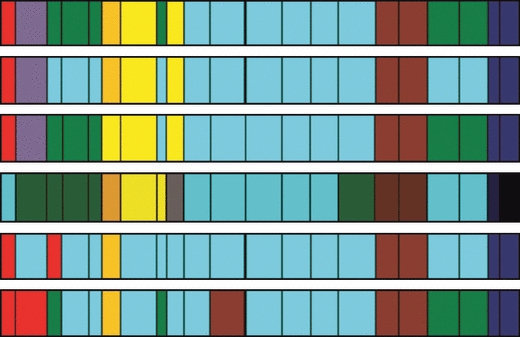
Clustering solutions of the three regions supported with the highest (top) and lowest (bottom) likelihood by the full data set. From top to bottom: clustering solutions of the regions Xr2, Xr4, 2r4, 3r3, 2r1 and 3r4. Populations order in the figure is the same as in Fig. 2.

Given that all regions differed significantly from each other, we compared them for their ability to recover the clustering solution of the complete data set. Hence, we calculated the marginal likelihood of the complete data set resulting in the clustering solution of each of the regions. The three regions with the highest marginal likelihood score were Xr2, Xr4 and 2r4. The smallest marginal likelihood score was obtained for regions 3r3, 2r1 and 3r4 (Table 4) with region 3r3 failing to reveal any population structure between the European, North-American, Australian, the Brazilian and the Asian samples (except Cebu) (Fig. 3).

**Table 4:** Support of each region’s data set to the full *Drosophila melanogaster* data set clustering solution. Results are ordered from top to bottom according to their marginal likelihood (the values refer to each clustering solution when applied to the full data set)

Clustering region	Log (marginal likelihood)
Xr2	−144 746.726
Xr4	−144 875.0135
2r4	−144 980.7091
2r5	−145 053.5297
3r2	−145 356.1026
4r2	−145 694.1783
3r5	−145 735.4679
Xr5	−145 886.1499
2r2	−146 170.216
Xr3	−146 426.5436
3r1	−146 530.5746
2r3	−146 550.7418
Xr1	−146 664.9901
2r1	−146 982.8241
3r3	−147 103.3378
3r4	−148 003.3915

Given the large heterogeneity in significant clustering solutions observed for the 16 regions analysed, we were interested whether some properties of the analysed regions affect the ability to recover the clustering of the complete data set. A wide range of explanatory variables (e.g. gene diversity, number of genes, inversions) was examined, but none of them could explain the clustering heterogeneity among regions (Table S5 and Fig. S5, Supporting information).

We further suspected that selective sweeps—restricted to local genomic regions—may have affected the partitioning of allelic variation among the populations, resulting in alternative clustering solutions for the different regions (Beisswanger *et al.* 2006; Turner *et al.* 2010). We tested this hypothesis by genotyping four additional microsatellites in two randomly selected regions with a clustering solution different from that of the full data set (Xr3 and Xr4). Other than expected under a scenario of selective sweeps, increasing the number of markers by 50% resulted in a significantly different clustering solution (Kolmogorov–Smirnov test, *P <*0.0005, Fig. S3, Supporting information) supported with a posterior probability higher than 0.98. Based on this result, we concluded that natural selection is not the cause for the different clustering solutions among genomic regions.

### Reliability of the clustering solution depends on the number of loci

As linkage disequilibrium does not extend beyond 2 kb in *D. melanogaster* (Miyashita & Langley 1988; Langley *et al.* 2000), we expect markers to behave independently even within the genomic regions analysed by us. This allowed us to randomly pick subsets of markers from the full *D. melanogaster* data set to test the effect of the number of loci on clustering solution.

**Fig. 4:**
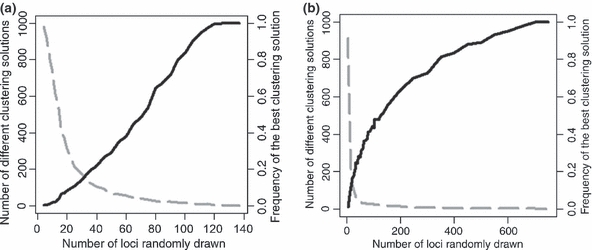
Change in frequency of the best clustering solution for different number of loci. The black solid line represents the change in frequency of the best clustering solution obtained with the complete data set. The grey dashed line represents the different number of clustering solutions for the different numbers of randomly sampled loci. (a) *Drosophila melanogaster* data set, (b) *Homo sapiens* data set.

Interestingly, data sets similar to those used for typical biogeographical surveys (i.e. comprising less than 30 loci) did not perform well—the probability of obtaining the globally best clustering solution was less than 15%. More than 95 loci were required to have 80% chance to capture the globally best clustering solution, and only when more than 120 markers were analysed, all data sets resulted in the best clustering solution. Similarly, we found that the total number of different clustering solutions obtained decreased with an increasing number of markers, indicating that the power to recover the best clustering solution depends on the number of markers analysed (Fig. 4a).

We repeated this analysis using a human data set representing 54 populations and 783 polymorphic microsatellites (Romero *et al.* 2009). Similarly to the *D. melanogaster* data set, we found that a large number of microsatellites need to be analysed to have high confidence in the obtained clustering solution. To obtain 95% confidence on the assignation of the populations to the five clusters described in the literature, 600 microsatellites are needed (Fig. 4b). Like for *D. melanogaster* up to 91.3% of the clustering solutions differed from each other when only five markers where randomly sampled, and the number of inconsistent clustering solutions rapidly dropped when larger sets of loci were analysed (Fig. 4b).

We complemented our result by computer simulations. We simulated 137 independent loci with ms using simulation parameters that coarsely matched the patterns of differentiation and variability in natural *D. melanogaster* populations (Begun & Aquadro 1993; Caracristi & Schlötterer 2003; Nunes *et al.* 2008). Like the real *D. melanogaster* data set, we found that a moderate number of loci frequently resulted in statistically well-supported clustering solutions that did not match the simulated demography. Only with a large number of loci, the true clustering could be recovered (i.e. 105 loci are needed to reach a 95% reliability on the clustering solution).

### Genealogical lineage sorting

So far, we demonstrated that a large number of loci are required to recover the genealogy of populations by using a Bayesian clustering method. We did not provide an explanation for the counter-intuitive observation of highly supported, but incorrect clustering solutions. The key insight explaining this result is depicted in Fig. 1. Genetic drift after the split of populations results in a stochastic lineage sorting, with some alleles being under-represented or even lost in one population, while highly frequent in another one. Unlinked loci will capture independent realizations of the drift process, possibly resulting in a different grouping of populations (Fig. 1).

**Fig. 5:**
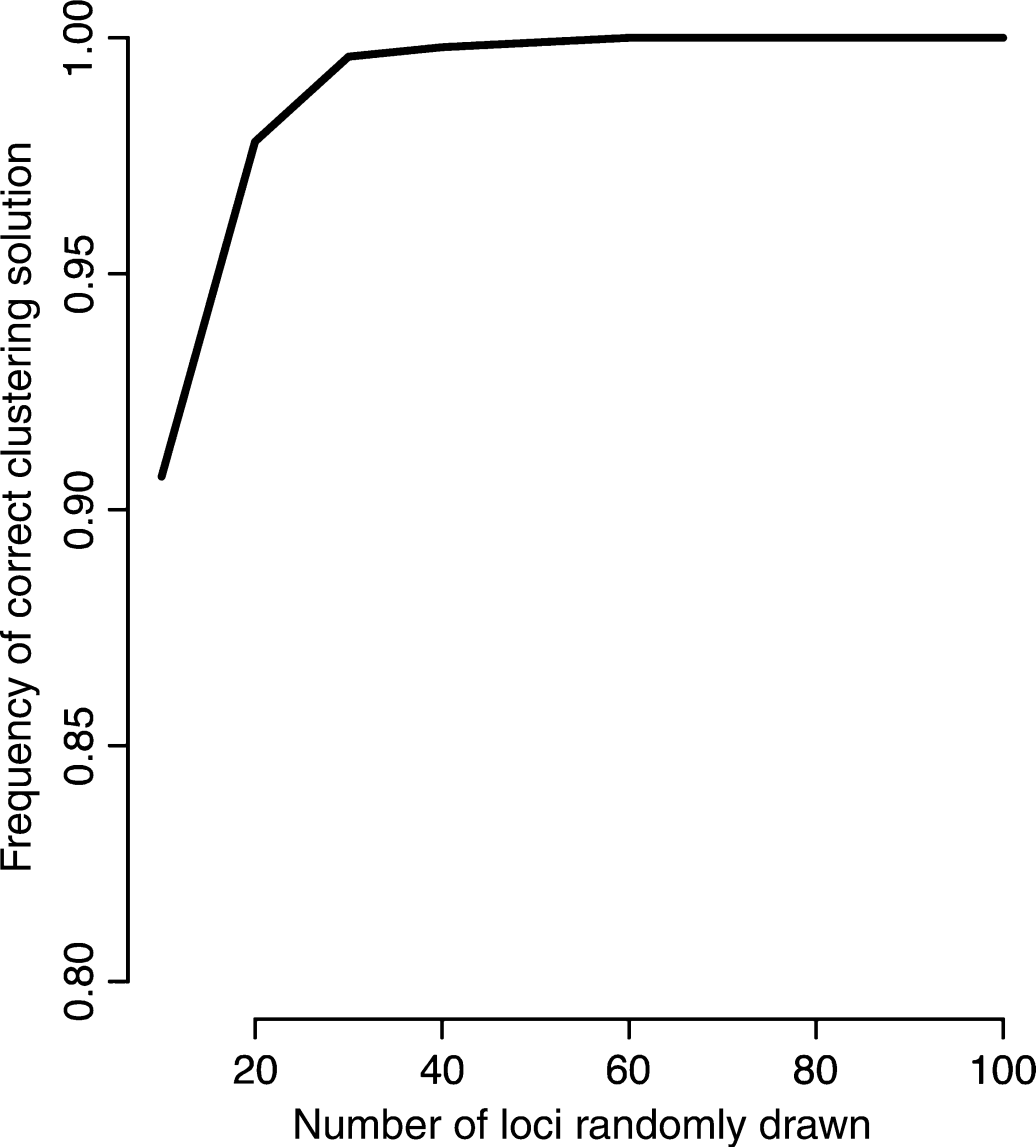
Simplified illustration of the effect of genealogical lineage sorting. The black line represents the frequency with which the true clustering solution [i.e. A(B,C)] occurs among 1000 random draws of a set of *n* loci (*n* from 10 to 100). The distribution of marker classes used to perform the random draws of *n* loci for this figure consisted of 50% of the loci reflecting the true clustering solution and an even proportion of the other types of loci resulting in different clustering solutions than the true one. The results based on other distributions of marker classes are shown in Data S1, Supporting information.

To illustrate how genealogical lineage sorting could result in the counter-intuitive result of a statistically well supported, but incorrect clustering solution, we analysed a scenario where three populations (A, B and C) recently diverged (Fig. 1) and were genotyped for five classes of loci. The classes result in either a clustering solution reflecting the correct pattern of population divergence [i.e. class 1 = A(B,C)] or in wrong clustering solutions [classes 2–5 = other clustering solutions than A(B,C)]. Using a majority rule, the results of this example recapitulate the observations with the real and simulated data sets (Figs 5 and S6, Supporting information), i.e. with a small number of loci, it is possible to obtain the incorrect clustering solution simply by chance as the majority of the sampled loci supports a wrong clustering solution. Furthermore, if the proportion of loci supporting the correct population structure is small relative to the proportions of other loci resulting in different clustering solutions (e.g. dashed blue line in Fig. S6, Supporting information), the probability of retrieving the true population structure does not increase despite the larger number of loci sampled.

**Fig. 6:**
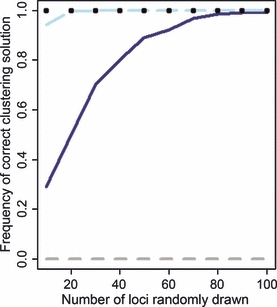
Relationship between population differentiation and genealogical inference. We used computer simulations to determine the frequency of correctly inferred clustering solutions in relationship to the number of loci used and population differentiation. Results are shown for *F*_ST_ values of: 0.01 (dashed grey line), 0.05 (solid blue line), 0.1 (dashed light blue line) and 0.15 (dotted black line).

We performed coalescent simulations to test how divergence among populations affects the genealogical lineage sorting. We assumed a simple demography with five populations branching off at the same time in the past. Using different numbers of loci and time points of the population split, we evaluated the clustering solutions. Consistently with previous results (Latch *et al.* 2006), very low differentiation (*F*_ST_ ≤ 0.01) always resulted in a single population cluster with high statistical support. On the contrary, high differentiation (*F*_ST_=0.1) led to the inference of the correct number of populations, even with a small number of loci (e.g. 30). Intermediate levels of differentiation (*F*_ST_ 0.01–0.05) showed the effect of genealogical lineage sorting, where for a low number of loci (e.g. 30), fewer than five populations were detected and supported with posterior probabilities larger than 0.95 (Fig. 6). This suggests that with intermediate levels of population differentiation, by chance the allele frequencies for two (or more) populations have not diverged to an extent that would allow distinguishing these populations as separate units. Most important, this should not be confounded with insufficient statistical power, as the posterior probability was generally high. Interestingly, we obtained qualitatively similar results when we used structure (Pritchard *et al.* 2000) rather than baps with a smaller data set (50 replicates for each draw of *n* loci in each *F*_ST_ scenario; Fig. S7, Supporting information). This suggests that the variation in inferences over sets of loci is not simply a consequence of the estimation algorithms used by baps, but a more common feature of Bayesian clustering–based inference in this context, which reflects true stochastic variation in biological signals.

## Discussion

This study reports the most comprehensive microsatellite data set (137 loci) in *Drosophila melanogaster* covering all chromosomes in 21 populations. With a significantly larger number of loci analysed than other worldwide microsatellite surveys in *D. melanogaster*, we recover features of the global pattern of genetic variation and divergence known in the species.

A distinct feature of this data set is that microsatellites were not randomly distributed over the genome, but clustered in 16 groups of markers within intervals of 83.3 (±16) kb. The naïve expectation would be that each group captures the same genealogy—or a slight modification of it, which statistically is not significantly different from the true underlying genealogy. Our analysis revealed, however, that all groups of markers resulted in highly supported clustering solutions (pp > 0.9), which nonetheless differed significantly from each other. Only a single group of eight markers resulted in the same clustering solution as the full data set. Strikingly, this heterogeneity in clustering solutions cannot be attributed to different properties of the genomic regions. Based on the analysis of additional markers in the same regions and computer simulations, we conclude that the markers in a region are independent of each other. Consistent with this, our computer simulations showed that random subsets of unlinked markers also produced strongly supported clustering solutions that differed significantly from each other. Only when a large number of loci are analysed jointly, it is possible to accept the obtained clustering solution with high confidence.

Interestingly, this effect cannot be attributed to insufficient statistical power with fewer loci, as most clustering solutions have high statistical support. Rather, the better performance with more markers is probably the outcome of a lower weight given to loci supporting an alternative clustering solution. Unfortunately, there is currently no tool available that predicts the number of loci required to have confidence in the obtained clustering solution.

Our observation contrasts a previous study, which suggested that in *D. melanogaster* as few as four microsatellite loci are sufficient to recover the known population structure of the species if the most informative markers are used. When selecting markers randomly, 10 markers are enough for 93% correct population assignment (Rosenberg 2005). We think that this discrepancy largely stems from the fact that Rosenberg (2005) used fewer populations than we did. Nonetheless, our observation that an extensive number of loci are also required for data sets other than the *D. melanogaster* one is supported by previous findings (Takezaki & Nei 1996, 2008). Using 12 populations of the H971 data set (*Homo sapiens*), the authors suggested that 500 microsatellites are required to obtain the expected tree topology with 95% certainty for average population sample sizes of 20 individuals. Furthermore, the results of Takezaki & Nei (2008) and our small computational experiments with structure software (see previous section) support the conclusion that these observations are not dependent on the method used (e.g. baps) but instead represent an intrinsic biological property of the data sets analysed.

Our results have important implications for the interpretation of clustering solutions, as many population surveys use only a moderate number of microsatellites to infer population structure. The naïve expectation is that too few loci should result in no evidence for population structure, rather than in a well-supported clustering solution that does not reflect the true population structure. Hence, statistically highly supported clustering solutions are currently presented in the literature as the true population structure without considering the important drift effects we have demonstrated in this report. However, it should also be kept in mind that the stochasticity in the population structure estimates as a function of the particular genomic regions surveyed will decrease when the level of genetic differentiation increases among the lineages. Thus, the problem is most accentuated for data sets showing low levels of genetic differentiation and gradually vanishes when the average differences between allele frequencies tend towards their maximum values. For strongly differentiated lineages, it is expected that even moderately sized random sets of informative loci will lead to highly concordant inferences about population structure (i.e. consistent clustering solutions).

One interesting example for the consequences of this is given by the comparison of population structure in *D. melanogaster* using X-linked and autosomal microsatellites (Schlötterer *et al.* 2006). One population from China grouped with Asian populations based on 23 X-linked microsatellites, but with European and American ones when using 26 markers on the second chromosome. In the data set of this study, we did not find evidence for a clustering with European and American populations when only second chromosomal microsatellites were used, suggesting that the results of Schlötterer *et al.* (2006) was an artefact of too few microsatellites used.

Based on our results, we advocate that authors should not only rely on probabilities obtained by clustering software, such as baps or structure, but use computer simulations, similar to the ones we used to obtain some power estimates about the number of loci needed to obtain reliable clustering solutions. In addition, measures of genetic differentiation, such as *F*_ST_, among the obtained clusters will also enable one to assess the expected stability of the population structure estimates for other sets of loci.

## Supporting information

**Data S1** Materials and methods.

**Table S1** *Drosophila melanogaster* Microsatellite primer pairs.

**Table S2** Populations used for the human population study.

**Table S3** Microsatellite used for the human population study.

**Table S4** Heterozygosities for the different chromosomes in African and Non-African *Drosophila melanogaster* populations.

**Table S5** Results of the linear models.

**Table S6** Relationship between the number of loci and divergence for the estimate of population structure.

**Fig. S1** Microsatelite marker design. Schematic of a chromosome where one region wherein microsatellites where genotyped has been magnified. The enlarged region shows eight marks in red which represent the microsatellite positions within the region. The X, 2nd and 3rd chromosomes have 5 such regions along their length and the 4th chromosome due to its small size only one.

**Fig. S2** Clustering solutions for each chromosome.

**Fig. S3** Clustering solutions for each genomic region. Each clustering solution is labeled with its correspondent region label.

**Fig. S4** Histograms of the ml-differences distribution for the regions against each of the chromosomes. (A) comparison for the X regions, (B) comparison for the 2nd chromosome regions, (C) comparison for the 3rd chromosome regions and (D) comparison for the region in the 4th chromosome.

**Fig. S5** Correlation plots of the region's properties against the region's ranking position. Log(ml): logarithm of the marginal likelihood of the regions' clustering solutions.

**Fig. S6** Simplified illustration of the effect of genealogical lineage sorting. Each line represents the frequency with which the true clustering solution [i.e. A(B,C)] occurs among 1000 random draws of a set of *n* loci (*n* from 10 to 100). The lines correspond to the results from drawing loci from a distribution of markers where the proportion of loci resulting in the true population structure is: 20% (dashed blue), 30% (dotted gray), 40% (dashed-dotted green), 50% (long-dashed orange), 60% (dashed red). When the loci that result in the true population structure occur with a frequency of 70% or higher in the genome 10 or more loci result in the expected clustering solution (solid-black line).

**Fig. S7** Relationship between population differentiation and genealogical inference. We used computer simulations to determine the frequency of correctly inferred clustering solutions in relationship to the number of loci used and population differentiation when using two Bayesian methods to infer population structure, i.e. BAPS and STRUCTURE. Results are shown for 50 simulations of 5 populations with average *F*_ST_ values of: 0.01 (dashed grey line), 0.05 (solid blue line) and 0.1 (dashed light blue line). For a detailed explanation of the simulated datasets see Data S1, Supporting information.
