# Supplementary material for: Genealogical lineage sorting leads to significant, but incorrect Bayesian multilocus inference of population structure
Source: Mol Ecol. 2011 Mar;20(6):1108–21. doi: 10.1111/j.1365-294X.2010.04990.x (PMC3084510; doi:10.1111/j.1365-294X.2010.04990.x)
Supplement: Supplementary file 1 [file mec0020-1108-SD1.doc]

Supporting information:

Materials & Methods

Kolmogorov-Smirnov tests:

As the Kolmogorov-Smirnov tests were calculated for pairs of sets of 10,000 observations each, a possible concern is that small differences between two otherwise identical distributions could result in significant test results. To corroborate our results we randomly extracted sets of 100 samples from the 10,000 values for different regions and repeated the analysis. The results did not change which indicated that the observed differences between the regions are real, and thus the clustering solutions are significantly different from each other.

Commands lines for ms.

The command used to generate the dataset reflecting the evolutionary history of 13 *D. melanogaster* populations is: ./ms 780 1000 -t 10 -I 13 60 60 60 60 60 60 60 60 60 60 60 60 60 -ej 0.04 13 1 -ej 0.0325 12 11 -ej 0.038 11 13 -ej 0.0385 3 13 -ej 0.030005 4 3 -ej 0.03 5 4 -ej 0.015 6 5 -ej 0.0132 7 6 -ej 0.012 8 7 -ej 0.0325 10 3 -ej 0.0012 9 10 -ej 0.0125 2 1 -en 0.039992 1 1.75 -en 0.039992 13 0.00001 -en 0.038008 13 1 -en 0.036 3 0.02 -en 0.03 13 0.05 -en 0.03 12 0.02 -en 0.03 11 0.02.

The commands used to generate the simulations of 5 populations that simultaneously diverge from their common ancestor are:

Average *F_ST_* 0.01: ./ms 500 1200 -t 1 -I 5 100 100 100 100 100 -ej 0.005 1 2 -ej 0.005 2 3 -ej 0.005 3 4 -ej 0.005 4 5

Average *F_ST_* 0.05: ./ms 500 1200 -t 1 -I 5 100 100 100 100 100 -ej 0.03 1 2 -ej 0.03 2 3 -ej 0.03 3 4 -ej 0.03 4 5

Average *F_ST_* 0.1: ./ms 500 1200 -t 1 -I 5 100 100 100 100 100 -ej 0.058 1 2 -ej 0.058 2 3 -ej 0.058 3 4 -ej 0.058 4 5

Average *F_ST_* 0.15: ./ms 500 1200 -t 1 -I 5 100 100 100 100 100 -ej 0.1 1 2 -ej 0.1 2 3 -ej 0.1 3 4 -ej 0.1 4 5

Supporting Table 1. *Drosophila melanogaster* Microsatellite primer pairs.

| Chromosome | Primer Pair Code | | sequence | Genomic position start | Genomic position end |
| --- | --- | --- | --- | --- | --- |
| X | XR1M1f | forward | ccagaaaaatgatcgagctg | 1939294 | 1939313 |
|  | XR1M1r | reverse | cttagcgattgcccactgt | 1939413 | 1939395 |
|  | XR1M2f | forward | atatgaggcacccaaagtgg | 1948425 | 1948444 |
|  | XR1M2r | reverse | cggctctttaacaatacgaatg | 1948679 | 1948658 |
|  | XR1M3f | forward | accagcggcaataaatgtct | 1954531 | 1954550 |
|  | XR1M3r | reverse | ggactatccgttccacatgc | 1954979 | 1954960 |
|  | XR1M4f | forward | tcgggaacctttccatacg | 1962519 | 1962537 |
|  | XR1M4r | reverse | ggcaaaagttagaatttctgga | 1962643 | 1962622 |
|  | XR1M5f | forward | gaacgcaaagagagcagagc | 1979418 | 1979437 |
|  | XR1M5r | reverse | ctatctgaccagggccactc | 1979679 | 1979660 |
|  | XR1M6f | forward | gcgtgtgcaagtgtgtgtaag | 1990927 | 1990947 |
|  | XR1M6r | reverse | gccatagcctcatctcgttc | 1991338 | 1991319 |
|  | XR1M7f | forward | ctgagccccacaaatgtttt | 1994306 | 1994325 |
|  | XR1M7r | reverse | aaccaaagcaaggcagagag | 1994452 | 1994433 |
|  | XR1M8f | forward | acgcaaaaacagatcgcatt | 2006780 | 2006799 |
|  | XR1M8r | reverse | aacgtacagcagccacagg | 2007054 | 2007036 |
|  | XR2M1f | forward | tggattttggatctgccttag | 4241724 | 4241744 |
|  | XR2M1r | reverse | gtgtcccgccctttgtagt | 4241869 | 4241851 |
|  | XR2M2f | forward | ttgaagattcgttattgctggt | 4253656 | 4253677 |
|  | XR2M2r | reverse | ctcggtggctattttgacg | 4253920 | 4253902 |
|  | XR2M3f | forward | gccaggatgcaaggactatg | 4267319 | 4267338 |
|  | XR2M3r | reverse | gctcaattacagtgcaacagaca | 4267758 | 4267736 |
|  | XR2M4f | forward | cttctacgccgccttttc | 4275784 | 4275801 |
|  | XR2M4r | reverse | ttgcgcttatttccactttc | 4275930 | 4275911 |
|  | XR2M5f | forward | aaaagagcctgtggaaccag | 4293993 | 4294012 |
|  | XR2M5r | reverse | ttgttggcagcttgttatgg | 4294281 | 4294254 |
|  | XR2M6f | forward | cttcgctcgcacacttaacc | 4308008 | 4308027 |
|  | XR2M6r | reverse | acaataagcagccgagatgg | 4308412 | 4308393 |
|  | XR2M7f | forward | cccgaaacaacaagaacagc | 4330693 | 4330712 |
|  | XR2M7r | reverse | ttgcagtgacatgaggtgttg | 4330837 | 4330817 |
|  | XR2M8f | forward | gggaagacgctcggtgac | 4345324 | 4345341 |
|  | XR2M8r | reverse | gcccccaaaagaaaagtgg | 4345590 | 4345572 |
|  | Xr3M1f | forward | atggcgtgggtgaaatg | 8573624 | 8573640 |
|  | Xr3M1r | reverse | tcaggacacaaaggcacat | 8573728 | 8573710 |
|  | Xr3M2f | forward | gaaggccatttcactgtcaag | 8592731 | 8592751 |
|  | Xr3M2r | reverse | gaatattggacttggggaatg | 8592862 | 8592842 |
|  | Xr3M3f | forward | catcgaatgcccaagctc | 8617227 | 8617244 |
|  | Xr3M3r | reverse | cagcgatctgttccacgtc | 8617394 | 8617376 |
|  | Xr3M4f | forward | tttcgcctgacccacatt | 8624629 | 8624646 |
|  | Xr3M4r | reverse | gcggcgattgcaatgat | 8624818 | 8624802 |
|  | Xr3M5f | forward | cgtcacacgtcaggacttt | 8600395 | 8600413 |
|  | Xr3M5r | reverse | cgtgtgctagtggcttgtct | 8600619 | 8600600 |
|  | Xr3M6f | forward | cgtgcgaagtgatgtgatgt | 8664774 | 8664793 |
|  | Xr3M6r | reverse | ctgttgttgcttccgctgt | 8665024 | 8665006 |
|  | Xr3M7f | forward | gccctcacacctcgaatatc | 8674780 | 8674799 |
|  | Xr3M7r | reverse | ctgcaaaccacaagggaac | 8675060 | 8675042 |
|  | Xr3M8f | forward | caaacagacacacccactcac | 8590623 | 8590643 |
|  | Xr3M8r | reverse | accgccctcgagtcattt | 8590836 | 8590819 |
|  | Xr3M10f | forward | agcgaacaaatttccacgta | 8631346 | 8631365 |
|  | Xr3M10r | reverse | gccttaaagggagttgtcatt | 8631570 | 8631550 |
|  | Xr3M11f | forward | gctaaagtaaatcgccgttcc | 8654624 | 8654644 |
|  | Xr3M11r | reverse | cccaccgctgagaagagtta | 8654815 | 8654796 |
|  | Xr3M12f | forward | gctgacttccgccaatctg | 8647215 | 8647233 |
|  | Xr3M12r | reverse | gccgcatttagcaacttca | 8647529 | 8647511 |
|  | Xr3M13f | forward | atcgtcaacagcagcaacat | 8579158 | 8579177 |
|  | Xr3M13r | reverse | ctctcaagctcacggctcat | 8579315 | 8579296 |
|  | Xr4M1f | forward | cgcatttgtgtcgcattg | 12495264 | 12495281 |
|  | Xr4M1r | reverse | aaagagcgggtgggagaga | 12495603 | 12495585 |
|  | Xr4M2f | forward | gcaacactgagaacacagacag | 12475044 | 12475065 |
|  | Xr4M2r | reverse | taaggtgacagcgagacagg | 12475417 | 12475398 |
|  | Xr4M3f | forward | cacccatttcgtccagtttc | 12488169 | 12488188 |
|  | Xr4M3r | reverse | acacattgaagcgcacagc | 12488272 | 12488254 |
|  | Xr4M4f | forward | gcaagcagttcagaaagagagg | 12460031 | 12460052 |
|  | Xr4M4r | reverse | gcgtcctttgcttggtctt | 12460313 | 12460295 |
|  | Xr4M5f | forward | atcgcccagctcagatca | 12474758 | 12474775 |
|  | Xr4M5r | reverse | ggttcactgcgattcggtta | 12474890 | 12474871 |
|  | Xr4M6f | forward | taacggtggctttgtgct | 12503674 | 12503691 |
|  | Xr4M6r | reverse | cgaggagagagaagaagagtgg | 12503837 | 12503816 |
|  | Xr4M7f | forward | aaagtctcagccgtaaagagagc | 12533506 | 12533528 |
|  | Xr4M7r | reverse | aatccaaccgaaagccatag | 12533695 | 12533676 |
|  | Xr4M8f | forward | atgcacacgcacacctgtc | 12513868 | 12513886 |
|  | Xr4M8r | reverse | tggaagacatcggaccaact | 12514087 | 12514068 |
|  | Xr4M9f | forward | accgcatacgcacacaac | 12483520 | 12483537 |
|  | Xr4M9r | reverse | gagcgaacgagcgagaaa | 12483828 | 12483811 |
|  | Xr4M10f | forward | gagggaaattcaacgaagaac | 12467270 | 12467290 |
|  | Xr4M10r | reverse | gggccaataggaaatatgc | 12467610 | 12467592 |
|  | Xr4M11f | forward | gccaatgcatcgtgacca | 12523957 | 12523974 |
|  | Xr4M11r | reverse | ccagtggtttcgcctatgg | 12524323 | 12524305 |
|  | Xr4M12f | forward | tgtaactctccgctgcttga | 12507754 | 12507773 |
|  | Xr4M12r | reverse | tttcattcccaccgtttctt | 12507977 | 12507958 |
|  | Xr5M1f | forward | tgagtgatcgcacactgctaa | 15907856 | 15907876 |
|  | Xr5M1r | reverse | caaaccgatatgctcacttacg | 15908105 | 15908084 |
|  | Xr5M2f | forward | gcctccgggttgaacatt | 15915137 | 15915154 |
|  | Xr5M2r | reverse | ctgtctgaatagaccgaaatagca | 15915449 | 15915426 |
|  | Xr5M3f | forward | cgtacactgcgtaggttatgaaa | 15937461 | 15937483 |
|  | Xr5M3r | reverse | gccttcaatacgggctcttc | 15937832 | 15937813 |
|  | Xr5M4f | forward | caactgaaagccatgcagaa | 15945293 | 15945312 |
|  | Xr5M4r | reverse | aagcaatgatgcggatttg | 15945635 | 15945617 |
|  | Xr5M5f | forward | gacttcgcgttcgaccag | 15965532 | 15965549 |
|  | Xr5M5r | reverse | gcgcattcaagtcaaagtca | 15965694 | 15965675 |
|  | Xr5M6f | forward | tgacaaagacatgccaatga | 15994968 | 15994987 |
|  | Xr5M6r | reverse | tcactgaacacgcgcataa | 15995157 | 15995139 |
|  | Xr5M7f | forward | tcacctttagaatcggcgtta | 15980797 | 15980817 |
|  | Xr5M7r | reverse | caacatacaaagcggagagc | 15981021 | 15981002 |
|  | Xr5M8f | forward | attccagcgtagcagtagca | 16002899 | 16002918 |
|  | Xr5M8r | reverse | cgtcccagagatagaaccaaac | 16003183 | 16003162 |
| 2 L | 2r1m1NEWf | forward | catcttcacttagcgcaacact | 1279674 | 1279695 |
|  | 2r1m1NEWr | reverse | ccttctggcggaatcctt | 1279955 | 1279938 |
|  | 2r1m2f | forward | accgccagatgccaaag | 1237572 | 1237588 |
|  | 2r1m2r | reverse | cgtcgaaaggtgagcgattt | 1237676 | 1237657 |
|  | 2r1m3f | forward | ttcgcccatccaactatt | 1245484 | 1245501 |
|  | 2r1m3r | reverse | gcggcgtaaataagtcataag | 1245648 | 1245628 |
|  | 2r1m4f | forward | tgcgaatggaagtcgagtaa | 1256688 | 1256707 |
|  | 2r1m4r | reverse | gtgcatgggtccacttgag | 1256942 | 1256924 |
|  | 2r1m5f | forward | aaagtcgcagagatggatgtt | 1243997 | 1244017 |
|  | 2r1m5r | reverse | aaccagccgcacagaca | 1244096 | 1244080 |
|  | 2r1m6f | forward | gatgcccaaccaagcaaat | 1270685 | 1270703 |
|  | 2r1m6r | reverse | gtcgaaatgcccgaagaaat | 1270875 | 1270856 |
|  | 2r1m7f | forward | tttcaacgcgcttctctc | 1278200 | 1278217 |
|  | 2r1m7r | reverse | actgatttgacaccaaagagc | 1278424 | 1278404 |
|  | 2r1m8f | forward | tcgcaagtctaatctgtgaatagc | 1229519 | 1229542 |
|  | 2r1m8r | reverse | ggagaacgtgagtagaaatatctgag | 1229831 | 1229806 |
|  | 2r2m1f | forward | ggcagagaatcgaaacgaa | 7087509 | 7087527 |
|  | 2r2m1r | reverse | cccttcgcgctacatacata | 7087670 | 7087651 |
|  | 2r2m2f | forward | tcccgaactgtgctaatgttt | 7088315 | 7088335 |
|  | 2r2m2r | reverse | gccaagaataaaggaggcataa | 7088539 | 7088518 |
|  | 2r2m3f | forward | acaggcgagcaaagcaac | 7094199 | 7094216 |
|  | 2r2m3r | reverse | tcagctcttcttcctaccaagttt | 7094331 | 7094308 |
|  | 2r2m4f | forward | ctcaccacccgtcccttac | 7096728 | 7096746 |
|  | 2r2m4r | reverse | ctgttccaaattgcgttattca | 7097007 | 7096986 |
|  | 2r2m5f | forward | gcaacttcaacaagcccatag | 7122045 | 7122065 |
|  | 2r2m5r | reverse | agtgcaatgacaacgggaac | 7122325 | 7122306 |
|  | 2r2m6f | forward | ctcacggacgcagatacaga | 7133965 | 7133984 |
|  | 2r2m6r | reverse | tgcagaggcagtagcaacat | 7134247 | 7134228 |
|  | 2r2m7New03f | forward | atttggcttgatgcggttt | 7110799 | 7110817 |
|  | 2r2m7New03r | reverse | aaagagcttggctgcaagaa | 7110992 | 7110973 |
|  | 2r2m8f | forward | agaataccagagcgcacaat | 7160865 | 7160884 |
|  | 2r2m8r | reverse | tttctggccctcttctagg | 7161029 | 7161011 |
|  | 2r3m1f | forward | ggcgtaatgccagacgaa | 12608158 | 12608175 |
|  | 2r3m1r | reverse | gctgtaacacgatacgggagt | 12608319 | 12608299 |
|  | 2r3m2f | forward | gggatcgtccaacagaagc | 12602661 | 12602679 |
|  | 2r3m2r | reverse | cagcagccaaggaagctaat | 12602881 | 12602862 |
|  | 2r3m3f | forward | caggccaacaacacttcaac | 12576285 | 12576304 |
|  | 2r3m3r | reverse | aaatctgacacatggcgaaa | 12576387 | 12576368 |
|  | 2r3m4f | forward | cagcatttcgcttgatttga | 12623836 | 12623855 |
|  | 2r3m4r | reverse | gctccatttagcacaaccaat | 12624087 | 12624067 |
|  | 2r3m5NEWf | forward | aatcgcagtacccattccac | 12568931 | 12568950 |
|  | 2r3m5NEWr | reverse | cggaggtaaaactttcttttcg | 12569244 | 12569223 |
|  | 2r3m6f | forward | agcgaaatcagaagacgacaa | 12617786 | 12617806 |
|  | 2r3m6r | reverse | cttgtttgaggcgtggctta | 12618039 | 12618020 |
|  | 2r3m7f | forward | tatggcccaggactccttc | 12654094 | 12654112 |
|  | 2r3m7r | reverse | ccgttatacacgggtgaagc | 12654287 | 12654268 |
|  | 2r3m8f | forward | tcaaggagcttgtcttgtaacg | 12658116 | 12658137 |
|  | 2r3m8r | reverse | gatctctatgatggattcagttcg | 12658369 | 12658346 |
| 2 R | 2r4m1f | forward | tttgcatttacaagaagtggtct | 5804822 | 5804844 |
|  | 2r4m1r | reverse | gccacaaccgaaatacatca | 5804953 | 5804934 |
|  | 2r4m2f | forward | tttccacccattctacacacc | 5825348 | 5825368 |
|  | 2r4m2r | reverse | acccaaagagagggaccaaa | 5825447 | 5825428 |
|  | 2r4m3NEWf | forward | tggaacccgatggataaact | 5806550 | 5806569 |
|  | 2r4m3NEWr | reverse | aactctttaaccgtgcttttgtt | 5806769 | 5806747 |
|  | 2r4m4f | forward | tatttaccgagcacgacgaa | 5831952 | 5831971 |
|  | 2r4m4r | reverse | cagcacgaatgaacactgaa | 5832236 | 5832217 |
|  | 2r4m5NEW02f | forward | tccgccagttagtctgtcaa | 5843150 | 5843169 |
|  | 2r4m5NEW02r | reverse | gagtccagttgcttgccatt | 5843369 | 5843350 |
|  | 2r4m6f | forward | aatttcggtgtcctcggatt | 5868772 | 5868791 |
|  | 2r4m6r | reverse | tttatcctgcctttgggttg | 5869111 | 5869092 |
|  | 2r4m7NEWf | forward | atctttcccactctccgatg | 5883145 | 5883164 |
|  | 2r4m7NEWr | reverse | cagcaaagagaagcaacaaca | 5883514 | 5883494 |
|  | 2r4m8f | forward | ggccaacgggagtgttaat | 5890927 | 5890945 |
|  | 2r4m8r | reverse | tctgccagcaactgaagc | 5891266 | 5891249 |
|  | 2r5m1f | forward | aataatacaacaggcggagctt | 17410142 | 17410163 |
|  | 2r5m1r | reverse | agccaatgcgaaaccaact | 17410242 | 17410224 |
|  | 2r5m2f | forward | gcacgcctctctaacctcaa | 17434536 | 17434555 |
|  | 2r5m2r | reverse | agtttcacattacgagccacaa | 17434696 | 17434675 |
|  | 2r5m3f | forward | gtgagcgaatggagagagca | 17401824 | 17401824 |
|  | 2r5m3r | reverse | tatcggcgtcgggtgatta | 17401953 | 17401935 |
|  | 2r5m4f | forward | ttctgttcacaatgaatgaggaat | 17404575 | 17404598 |
|  | 2r5m4r | reverse | cgaagtaatggaaacatagaactcaa | 17404769 | 17404744 |
|  | 2r5m5f | forward | aagtgccacacaacggtaagt | 17465138 | 17465158 |
|  | 2r5m5r | reverse | aatggcggcggaagtaag | 17465418 | 17465401 |
|  | 2r5m6f | forward | tgcaactgaagaagaagcagaa | 17467390 | 17467411 |
|  | 2r5m6r | reverse | gcagttcaaatcgcaactctc | 17467554 | 17467534 |
|  | 2r5m7f | forward | gtggtgccaggctaacaaa | 17472717 | 17472735 |
|  | 2r5m7r | reverse | atttgccagcgacggaat | 17472938 | 17472921 |
|  | 2r5m8f | forward | ggcagcaacggttagatttc | 17481433 | 17481452 |
|  | 2r5m8r | reverse | ggcctcagtacgcatgtacc | 17481742 | 17481723 |
| 3 L | 3r1M1Newf | forward | ttaatcagtcgcagtcccaac | 1579106 | 1579126 |
|  | 3r1MNew1r | reverse | tggcaataaatccaaacatctg | 1579268 | 1579247 |
|  | 3r1M2Newf | forward | gcactcactcaaatacactctcac | 1586450 | 1586473 |
|  | 3r1MNew2r | reverse | aagttatgccgatgcacttg | 1586553 | 1586534 |
|  | 3r1M3Newf | forward | tggattgactgggttgaatg | 1626490 | 1626509 |
|  | 3r1M3Newr | reverse | aaactaaggaaaccaactaaacgtact | 1626623 | 1626597 |
|  | 3r1M4Newf | forward | gattcctttgtggagccttg | 1629880 | 1629899 |
|  | 3r1M4Newr | reverse | cgtgcaaacagggtaatgg | 1630070 | 1630052 |
|  | 3r1M5Newf | forward | caatctcagacaacgatacacg | 1632519 | 1632540 |
|  | 3r1M5Newr | reverse | gaggctcaaaggaagacagc | 1632743 | 1632724 |
|  | 3r1M6Newf | forward | ggaagacagtggatattaggcttt | 1662503 | 1662526 |
|  | 3r1M6Newr | reverse | cgtaaccgatgaaattatagcaa | 1662817 | 1662795 |
|  | 3r1M7Newf | forward | tagagggttgcgctctcagt | 1637056 | 1637075 |
|  | 3r1M7Newr | reverse | gcccatttcaagcattaacc | 1637335 | 1637316 |
|  | 3r1M8New02f | forward | gaatcgagggaatcctgtagc | 1552864 | 1552884 |
|  | 3r1M8New02r | reverse | ccgtcgagtgcctaatacaaa | 1552966 | 1552946 |
|  | 3r2M1f | forward | tgtgtatgcccgatcatttg | 6919011 | 6919030 |
|  | 3r2M1r | reverse | gggctcctcctttcaacttc | 6919295 | 6919276 |
|  | 3r2M2fNew02 | forward | aaatgttgcaaatgttactcaacac | 6945701 | 6945725 |
|  | 3r2M2rNew02 | reverse | tgcattgctggttatatgatgtt | 6946037 | 6946015 |
|  | 3r2M3f | forward | gacgttcccacttcgtcgt | 6936039 | 6936057 |
|  | 3r2M3r | reverse | gggaaagccaatcggataa | 6936138 | 6936120 |
|  | 3r2M4f | forward | atagctggcagcgacgac | 6903595 | 6903612 |
|  | 3r2M4r | reverse | cagttgagcatttcttcagcac | 6903696 | 6903675 |
|  | 3r2M5f | forward | ccacattgctgctcttgttg | 6955527 | 6955546 |
|  | 3r2M5r | reverse | tgaatctaccagccacatacca | 6955901 | 6955880 |
|  | 3r2M6f | forward | aaagcgccaaatgtgagatg | 6960082 | 6960101 |
|  | 3r2M6r | reverse | ctaccgcctcctgttgtgac | 6960335 | 6960316 |
|  | 3r2M7f | forward | caacacgttgcaagttcgata | 6971449 | 6971469 |
|  | 3r2M7r | reverse | ggcgcctcatttgaaattct | 6971788 | 6971769 |
|  | 3r2M8f | forward | caaagcgcctgatctatggt | 6921808 | 6921827 |
|  | 3r2M8r | reverse | ctgctggatttggctgtgt | 6921996 | 6921978 |
|  | 3r3M1f | forward | cagagttcgtcatcgtcctgt | 13739677 | 13739697 |
|  | 3r3M1r | reverse | gttgccacaatcgcatacaa | 13739987 | 13739968 |
|  | 3r3M2f | forward | caacgaaacacacggctatac | 13711295 | 13711315 |
|  | 3r3M2r | reverse | cgactgtaaattgcgcttgt | 13711548 | 13711529 |
|  | 3r3M3f | forward | aaagcgtcagctcaaggact | 13718583 | 13718602 |
|  | 3r3M3r | reverse | tgtatatgttattagccgagagagg | 13718744 | 13718720 |
|  | 3r3M4f | forward | cccacacaactgtcctgtagat | 13763614 | 13763635 |
|  | 3r3M4r | reverse | gggtacatgggatgctgatt | 13763954 | 13763935 |
|  | 3r3M5f | forward | cggatgaagcctgcactt | 13774735 | 13774752 |
|  | 3r3M5r | reverse | ccacaaacacagacacttatgg | 13775019 | 13774998 |
|  | 3r3M6f | forward | tgcacaaatggagcgtttc | 13776066 | 13776084 |
|  | 3r3M6r | reverse | gcctgtccacactgaacctt | 13776380 | 13776361 |
|  | 3r3M7f | forward | gaagagcacaggcgattgat | 13791624 | 13791643 |
|  | 3r3M7r | reverse | accttccgccaagcaaac | 13791753 | 13791736 |
|  | 3r3M8f | forward | cgggaaatgatgaaccaaat | 13794588 | 13794607 |
|  | 3r3M8r | reverse | ggcatacggcattctacctatc | 13794749 | 13794728 |
| 3 R | 3r4M1f | forward | atcgatggccgcacaaat | 13062204 | 13062221 |
|  | 3r4M1r | reverse | tccgagctgaaagcattgac | 13062514 | 13062495 |
|  | 3r4M2f | forward | cacagacaaacaggatcaaacag | 13068859 | 13068881 |
|  | 3r4M2r | reverse | atcgcgaaatgccacaga | 13068963 | 13068946 |
|  | 3r4M3f | forward | caaacacccatcggtattaacc | 13082858 | 13082879 |
|  | 3r4M3r | reverse | gcctttgtttggcgttgtta | 13083082 | 13083063 |
|  | 3r4M4f | forward | agcgtgtttgtcacacatatcc | 13084982 | 13085003 |
|  | 3r4M4r | reverse | gagctccagacgacgagtg | 13085355 | 13085337 |
|  | 3r4M5f | forward | cgttgcaggggacttgatt | 13105576 | 13105594 |
|  | 3r4M5r | reverse | aagtttgcgacccgcact | 13105799 | 13105782 |
|  | 3r4M6f | forward | gtttgccttactagaagcttgatg | 13109374 | 13109397 |
|  | 3r4M6r | reverse | ccagcactacaagggatctattc | 13109566 | 13109544 |
|  | 3r4M7f | forward | aaatgcgctcccagctaaat | 13124630 | 13124649 |
|  | 3r4M7r | reverse | cctcgatgggtaaccttgaa | 13124763 | 13124744 |
|  | 3r4M8f | forward | gccataaacaactgaacactgc | 13140406 | 13140427 |
|  | 3r4M8r | reverse | gggccctgaatacgagaata | 13140538 | 13140519 |
|  | 3r5M1f | forward | tgggtctgcgactcaaa | 23528849 | 23528865 |
|  | 3r5M1r | reverse | gttagccggccctagatact | 23529038 | 23529019 |
|  | 3r5M2f | forward | gggcgcgagtgctactt | 23543935 | 23543951 |
|  | 3r5M2r | reverse | gccgaatggctgagtatgaa | 23544279 | 23544260 |
|  | 3r5M3f | forward | caagcggaccgagcaatac | 23562297 | 23562315 |
|  | 3r5M3r | reverse | ttccactggcaaacttgttg | 23562396 | 23562377 |
|  | 3r5M4f | forward | gcacaagcgcacacaaata | 23566126 | 23566144 |
|  | 3r5M4r | reverse | aacccttagccgaattagca | 23566380 | 23566361 |
|  | 3r5M5f | forward | cacgatttgggtttcaaaga | 23589082 | 23589101 |
|  | 3r5M5r | reverse | atggattcccacatgcctta | 23589181 | 23589162 |
|  | 3r5M6f | forward | tagcgcaaatatcgagctgt | 23596340 | 23596359 |
|  | 3r5M6r | reverse | cctaactttcaacgactgctacac | 23596620 | 23596597 |
|  | 3r5M7f | forward | cccagcaggcaggagac | 23592460 | 23592476 |
|  | 3r5M7r | reverse | cattagtatcgcgctgcattt | 23592830 | 23592810 |
|  | 3r5M8f | forward | atacctggcggggagtctat | 23600111 | 23600130 |
|  | 3r5M8r | reverse | acccattgtgtcgctgatatt | 23600270 | 23600250 |
| 4 | 4r2m1f | forward | agaggcatcgccaaataaag | 366587 | 366606 |
|  | 4r2m1r | reverse | gcccactagtgtacatcttactaacc | 366901 | 366876 |
|  | 4r2m2f | forward | aagtgagtcaacaaacttgctctg | 377812 | 377835 |
|  | 4r2m2r | reverse | tgacttccctgacgtacatacata | 378156 | 378133 |
|  | 4r2m3f | forward | tttgaggaaattcagtgctttg | 380664 | 380685 |
|  | 4r2m3r | reverse | tgattctcggcctgaataatatg | 381038 | 381016 |
|  | 4r2m4f | forward | tgacgttgatcgcgagttt | 389499 | 389517 |
|  | 4r2m4r | reverse | tgccgaaaatgtagcagatg | 389689 | 389670 |
|  | 4r2m5New02f | forward | cttatttcatccgagcgattg | 406733 | 406753 |
|  | 4r2m5New02r | reverse | cccggtaaacataattgactatcag | 406926 | 406902 |
|  | 4r2m6f | forward | ggacagacaaacagggtcaga | 433422 | 433442 |
|  | 4r2m6r | reverse | cccaccaataaggctcaaca | 433795 | 433776 |
|  | 4r2m7New02f | forward | tcggtctataaagccaatgtga | 448657 | 448678 |
|  | 4r2m7New02r | reverse | tgccaattccgaaaatgtgt | 448906 | 448887 |
|  | 4r2m8f | forward | tacatagatgacatatgaggaattgaa | 449382 | 449408 |
|  | 4r2m8r | reverse | aaacattcttggtcatggtcatt | 449721 | 449699 |
|  | 4r2m9f | forward | agtgaagccaccgagatcc | 467032 | 467050 |
|  | 4r2m9r | reverse | agctagacacaaagcaatgcag | 467371 | 467350 |

Supporting Table 2. Populations used for the human population study.

Orcadian, Adygei, Russian, Basque, French, Italian, Sardinian, Tuscan, Mozabite, Bedouin, Druze, Palestinian, Balochi, Brahui, Burusho, Hazara, Kalash, Makrani, Pathan, Sindhi, Melanesian, Papuan, Colombian, Karitiana, Maya, Pima, BantuKenya, BantuSW, BantuSE, Mandenka, Yoruba, BiakaPygmy, MbutiPygmy, San, Han, Han-NChina, Dai, Daur, Hezhen, Lahu, Miao, Oroqen, She, Tujia, Tu, Xibo, Yi, Mongola, Naxi, Uygur, Cambodian, Japanese, Yakut and Surui.

Supporting Table 3. Microsatellite used for the human population study.

| D12S1638 |
| --- |
| D14S1007 |
| D9S1779 |
| D9S1825 |
| D7S2477 |
| D17S784 |
| D16S403 |
| D3S1262 |
| D10S189 |
| D20S103 |
| D8S261 |
| D8S262 |
| D8S560 |
| D4S403 |
| D5S408 |
| D4S408 |
| D16S3401 |
| D18S1390 |
| D8S503 |
| D10S212 |
| D1S235 |
| D11S969 |
| D3S1560 |
| D20S851 |
| D6S305 |
| D15S165 |
| D16S422 |
| D3S1311 |
| D1S2682 |
| D15S128 |
| D1S468 |
| D3S3630 |
| D2S2986 |
| D9S1838 |
| D13S285 |
| D3S3644 |
| D22S1169 |
| D9S1871 |
| D16S516 |
| D4S3360 |
| D6S2522 |
| D18S843 |
| NA-D10S-2 |
| D3S2409 |
| D6S1021 |
| D9S910 |
| D14S592 |
| D11S1993 |
| D5S2488 |
| D10S1221 |
| D10S1222 |
| D3S2418 |
| D6S1027 |
| D5S1480 |
| D18S858 |
| D15S652 |
| D10S1225 |
| D1S1627 |
| D12S2070 |
| D4S2394 |
| D13S779 |
| D12S1042 |
| D4S2397 |
| D2S1352 |
| D21S1440 |
| D2S1353 |
| D6S1031 |
| D15S655 |
| D12S1045 |
| D10S1230 |
| D1S3462 |
| D14S599 |
| D4S2361 |
| D10S1412 |
| D11S2362 |
| NA-D11S-1 |
| D3S4523 |
| D22S1045 |
| D16S748 |
| D16S2616 |
| NA-D1S-4 |
| D17S2193 |
| D18S1370 |
| D1S3720 |
| D1S1589 |
| D2S1356 |
| NA-D5S-1 |
| D16S3396 |
| D17S2195 |
| D9S2157 |
| D10S1208 |
| NA-D13S-1 |
| NA-D17S-1 |
| NA-D1S-1 |
| D18S1357 |
| NA-D18S-2 |
| D11S4459 |
| D6S1006 |
| D17S2180 |
| NA-D8S-2 |
| D17S1298 |
| NA-D7S-1 |
| D4S1625 |
| D1S1728 |
| D4S3243 |
| D10S2470 |
| D2S2952 |
| D11S4463 |
| D7S3046 |
| D7S3047 |
| D18S542 |
| D5S1456 |
| D22S683 |
| D16S539 |
| D13S1807 |
| D21S1432 |
| D6S2410 |
| D4S1644 |
| D2S1360 |
| NA-D10S-1 |
| NA-D1S-2 |
| NA-D1S-5 |
| D3S4529 |
| D20S1143 |
| D21S2052 |
| D1S3721 |
| D1S534 |
| D11S2363 |
| D2S427 |
| D18S535 |
| NA-D1S-3 |
| D5S2845 |
| D14S1426 |
| D7S3051 |
| NA-D16S-1 |
| D7S817 |
| D5S2849 |
| D8S2324 |
| NA-D8S-1 |
| D15S1507 |
| D6S2439 |
| D3S4545 |
| D6S2436 |
| D14S1434 |
| D2S2972 |
| D18S1371 |
| NA-D18S-1 |
| D2S2968 |
| NA-D6S-1 |
| D18S1376 |
| D17S2196 |
| NA-D9S-1 |
| D21S2055 |
| D7S3070 |
| NA-D14S-1 |
| D15S1515 |
| NA-D22S-1 |
| D8S1128 |
| D5S1457 |
| D22S689 |
| D9S922 |
| D19S1034 |
| D1S1594 |
| D16S3253 |
| D3S2427 |
| D4S2366 |
| D3S2387 |
| D19S586 |
| D13S787 |
| D2S1363 |
| D11S1998 |
| D7S1799 |
| D11S1999 |
| D6S1040 |
| D7S1818 |
| D7S3056 |
| D4S2367 |
| D17S1299 |
| D12S1052 |
| D8S1132 |
| D1S1596 |
| D15S642 |
| D9S925 |
| D2S1328 |
| D3S2432 |
| D1S1597 |
| D4S2368 |
| D11S2000 |
| D17S1301 |
| D4S3248 |
| D6S1959 |
| D1S3669 |
| D19S589 |
| D20S477 |
| D4S1647 |
| D5S816 |
| D14S606 |
| D7S3058 |
| D2S2944 |
| D11S2002 |
| D6S474 |
| D7S2846 |
| D14S1280 |
| D6S1009 |
| D7S1824 |
| D12S2078 |
| D3S1744 |
| D5S817 |
| D7S820 |
| D3S1763 |
| D5S1462 |
| D8S1136 |
| D7S1802 |
| D20S478 |
| D16S764 |
| D4S2417 |
| D1S1653 |
| D7S1804 |
| D14S608 |
| D13S793 |
| D19S591 |
| D20S480 |
| D11S2006 |
| D20S481 |
| D1S1660 |
| D9S930 |
| D11S1981 |
| D17S1290 |
| NA-D12S-1 |
| D3S1764 |
| D2S1334 |
| D2S410 |
| D2S434 |
| D12S395 |
| D12S372 |
| D1S549 |
| NA-D15S-1 |
| D8S1108 |
| D1S1609 |
| D15S643 |
| D13S796 |
| D20S482 |
| D2S1384 |
| D5S1501 |
| D9S2169 |
| D15S659 |
| D12S1064 |
| D11S2365 |
| D10S1239 |
| D6S1053 |
| D11S4464 |
| D13S800 |
| D9S934 |
| D18S877 |
| D2S1391 |
| D19S714 |
| D5S2500 |
| D5S2501 |
| D3S2460 |
| D6S1056 |
| D2S1394 |
| D1S551 |
| D8S592 |
| D11S1392 |
| D12S373 |
| D18S851 |
| D5S820 |
| D3S1766 |
| D3S2398 |
| D21S1446 |
| NA-D4S-1 |
| D10S1423 |
| D10S1425 |
| D2S1776 |
| D9S1118 |
| D16S2621 |
| D16S769 |
| D4S2632 |
| D2S1780 |
| D1S2134 |
| D3S3038 |
| D7S2204 |
| D10S1426 |
| D15S816 |
| D12S1294 |
| D14S742 |
| D1S518 |
| D5S1470 |
| D4S1627 |
| D9S301 |
| D18S1364 |
| D3S3039 |
| D8S1179 |
| D13S317 |
| D6S1277 |
| D9S1120 |
| D16S2624 |
| D3S3045 |
| D10S1430 |
| D5S2505 |
| D12S1300 |
| D15S818 |
| D2S1788 |
| D13S894 |
| D9S1121 |
| D10S1432 |
| D10S1435 |
| D2S1790 |
| D15S822 |
| D9S1122 |
| D5S1725 |
| D4S1629 |
| D20S201 |
| D3S1768 |
| D17S974 |
| D3S1746 |
| D2S441 |
| D2S405 |
| D8S1110 |
| D11S2371 |
| D12S1301 |
| D14S587 |
| D22S686 |
| D11S1984 |
| D4S2431 |
| D8S1477 |
| D2S1399 |
| D2S1400 |
| D14S617 |
| D9S938 |
| D13S895 |
| D1S1677 |
| D10S1248 |
| D13S1493 |
| D19S433 |
| D10S677 |
| D1S1612 |
| D21S1437 |
| D7S1808 |
| D16S753 |
| D14S588 |
| D3S2403 |
| D1S1679 |
| D7S3061 |
| D11S1986 |
| D8S1113 |
| D17S1294 |
| D10S2327 |
| D6S1017 |
| D17S1308 |
| D5S211 |
| D19S246 |
| D19S245 |
| D19S254 |
| D12S269 |
| D7S559 |
| D22S345 |
| NA-D12S-2 |
| F13A1-D6S |
| TPO-D2S |
| D20S159 |
| D21S1411 |
| D20S164 |
| D11S1304 |
| D20S451 |
| D12S297 |
| D6S942 |
| D8S1048 |
| D22S532 |
| D8S373 |
| D19S559 |
| D4S1652 |
| D7S821 |
| D1S1665 |
| D6S1051 |
| D5S1505 |
| D4S2623 |
| GATA194H05Z_1 |
| GTTTT002P_1 |
| GGAA23C07_1 |
| CATC015_1 |
| MFD424-TTTA003_1 |
| GATA23G09_1 |
| AAT238_1 |
| TTTA063P_1 |
| ATA008_1 |
| ATA43C09M_1 |
| ATA20F08P_1 |
| AGAT135_1 |
| AAT267_1 |
| AGAT017_1 |
| AAT258_1 |
| GATA2B02Z_1 |
| AGAT143_1 |
| GATA152F05L_1 |
| GATA193D02_1 |
| AAT259_1 |
| GATA113H03_1 |
| AAT252_1 |
| GGAA20F08_1 |
| GATA13C08M_1 |
| AGAT119M_1 |
| ATA73A08M_1 |
| ATA38A05_1 |
| TATC028_1 |
| AGAT118_1 |
| AAT200_1 |
| GATA51H01_1 |
| GATA135F02P_1 |
| AGAT141_1 |
| GATA88F03P_1 |
| GAT004_1 |
| ATA2E04_1 |
| GATA64F05_1 |
| ATA009_1 |
| AAT243_1 |
| AATA011_1 |
| TTTA049_1 |
| GATA28H06_2 |
| GAAT1A5_2 |
| AAT055ZM_2 |
| GATA056_2 |
| AAT263P_2 |
| AGAT117_2 |
| GATA194B06P_2 |
| ATA47C04P_2 |
| GATA174G01_2 |
| GATA10H05_2 |
| GATA130A05M_2 |
| GATA66D01_2 |
| GATA181G08M_2 |
| GATA70F12M_2 |
| ATA16D09_2 |
| GATA148G10P_2 |
| AAAT105ZP_2 |
| GATA139A04P_2 |
| TAGA002M_2 |
| GATA8H05_2 |
| AGAT093_2 |
| GATA63F01_2 |
| GATA43F06_2 |
| GATA126A06M_2 |
| GATA91D12M_2 |
| GATA194A05M_2 |
| ATA29E07M_2 |
| GATA149B10M_2 |
| GATA29A06M_2 |
| AAT203_2 |
| GATA23A02_2 |
| AGAT021_2 |
| MFD433-AGAT010_3 |
| TATC046_3 |
| GATA131D09_3 |
| ATCT053P_3 |
| GATA178C11M_3 |
| GATA146B10_3 |
| AAT264M_3 |
| TAT024Z_3 |
| GATA87B02_3 |
| AGAT128_3 |
| AAC023_3 |
| GGAT2G03_3 |
| GATA146D07_3 |
| ATC3D09_3 |
| GATA68D03_3 |
| ATC4D07_3 |
| TTTA033_3 |
| GATA152F04M_3 |
| ATA85B10P_3 |
| AAT071_3 |
| ATA21H07M_3 |
| GATA92B06P_3 |
| GATA72A06_3 |
| AAC030_3 |
| TTTA040_3 |
| ATA57D10M_3 |
| GGAA22B10P_3 |
| CTAT012_3 |
| MFD427-AAAT028_3 |
| AAT246_4 |
| TTTAT002Z_4 |
| ATT015_4 |
| TAAAA006_4 |
| ATCT018_4 |
| ATA21F01_4 |
| GATA29_4 |
| GAAT1F09P_4 |
| TAGA006_4 |
| GATA30B11_4 |
| GATA135C03M_4 |
| GATA150B10_4 |
| ATA44F05P_4 |
| GATA138G03_4 |
| TAGA049_4 |
| AATA045_4 |
| ATT077P_4 |
| GATA129D03M_4 |
| AGAT099P_5 |
| GATA31H11P_5 |
| AAAT111_5 |
| AAT248P_5 |
| ATAG078P_5 |
| GATA135G01_5 |
| GATA12A08P_5 |
| GATA63C02_5 |
| ATAG022_5 |
| AGAT030P_5 |
| GATA141B10M_5 |
| GATA138B05_5 |
| TAGA010_5 |
| GATA142H05P_5 |
| GATA51A07P_5 |
| GATA12G02_5 |
| AGAT122_5 |
| GATA73D11P_5 |
| AAT255_5 |
| AGAT130_5 |
| GATA51D11P_5 |
| ATA092P_5 |
| GATA139B09P_5 |
| AGAT126_5 |
| AAT266_5 |
| AAT013_5 |
| AAAT072_5 |
| ATT079P_5 |
| ATA109H09_6 |
| TTA032Z_6 |
| ATTT030_6 |
| AAT256P_6 |
| ATA50C05_6 |
| ATA12D05P_6 |
| GATA129G03P_6 |
| ATC033_6 |
| TAAA014P_6 |
| GATA29C09P_6 |
| GATA11B08P_6 |
| GATA112F02P_6 |
| GATA30A08M_6 |
| GATA136F05P_6 |
| GATA161A04P_6 |
| ATA38D08P_6 |
| ATA1F08_6 |
| TATC050ZM_6 |
| ATA6C09P_6 |
| TAT026P_6 |
| ATGA020_6 |
| AGC001B_6 |
| GATA61G06_7 |
| TATC010P_7 |
| TATT019_7 |
| GATA137A12M_7 |
| AGAT103_7 |
| GATA026M_7 |
| ATA31F09M_7 |
| TAT028P_7 |
| GATA4E04_7 |
| GATA21D12P_7 |
| GATA87D11_7 |
| ATA78C09ZP_7 |
| ATAC037P_7 |
| TTTA001M_7 |
| AGAT133_7 |
| ATA55A05_7 |
| GATA145G10M_7 |
| GATA63F08P_7 |
| AGAT049P_7 |
| TATG002P_7 |
| MFD442-GTTT002_7 |
| ATT023_8 |
| ATAA009_8 |
| TTCA004P_8 |
| GATA25C10M_8 |
| ATT070_8 |
| TATC012_8 |
| ATAA018P_8 |
| TTA024_8 |
| AATA019_8 |
| GATA156H01M_8 |
| ATAG042_8 |
| AAT076_8 |
| GATA3H11_8 |
| AGAT115_8 |
| AAAT121P_8 |
| TATC059_8 |
| GATA060_8 |
| MFD455-AAT052_9 |
| TAA005_9 |
| AAAAC001_9 |
| GATA175H06M_9 |
| AGAT142P_9 |
| TCTA020_9 |
| GATA165A11M_9 |
| ATA65H08P_9 |
| ATA18C09P_9 |
| GATA5E06P_9 |
| GATA61F04_9 |
| AGAT140P_9 |
| GATA22H04M_9 |
| GGAT3G09M_9 |
| AAT261_9 |
| AAAT126_9 |
| GATA27Z_9 |
| ATA42G04P_9 |
| GATA65D11_9 |
| TCTA017M_9 |
| AGAT125_9 |
| CTAT016_9 |
| TTTTA002_9 |
| ATAG053P_10 |
| ATA84D02_10 |
| ATCC001_10 |
| ATGT009P_10 |
| ATAG055_10 |
| GATA179E06P_10 |
| ATA20B07_10 |
| TCTA021ZM_10 |
| GATA90G05P_10 |
| GATA196C10P_10 |
| GATA134F03P_10 |
| GATA81F06_10 |
| ATA80B10Z_10 |
| TTA007M_10 |
| GATA88F08P_10 |
| ATA44G07M_10 |
| AAC1C02_10 |
| TACA003_10 |
| ATGT006Z_10 |
| AAT224M_11 |
| GATA73B08M_11 |
| ATA25D12_11 |
| GATA29B11_11 |
| AAT268_11 |
| TTA008P_11 |
| AAT265M_11 |
| TCTA025_11 |
| GATA35_11 |
| GATA71E06_11 |
| AAT228_11 |
| TAA003_11 |
| ATA27C11_11 |
| GATA101G01_12 |
| GATA22H02_12 |
| AAT262_12 |
| GATA7F09_12 |
| ATAAC002_12 |
| AAC040Z_12 |
| GATA91H01_12 |
| AAT142M_12 |
| ATA73C05P_12 |
| GATA167C12_12 |
| AGAT084_12 |
| GGAT2G06M_12 |
| AGAT114_12 |
| ATA10H03_12 |
| GGAA19H02_12 |
| ATA63A05P_12 |
| GGAA22C05_12 |
| GATA5H03_12 |
| ATA080M_12 |
| AAT253P_12 |
| AAAAT002_12 |
| AAAT134_13 |
| GATA86B09P_13 |
| AGAT110P_13 |
| GATA6B07_13 |
| GATA73A05_13 |
| AACAT001_13 |
| GATA137B09_13 |
| ATA38B10_13 |
| ACT3F12_13 |
| GATA100E02P_13 |
| GATA135E01P_13 |
| AGAT113Z_13 |
| GTT035_13 |
| ATA70B03P_14 |
| ATA77F05_14 |
| TCTA023P_14 |
| AGAT116P_14 |
| GATA136A04_14 |
| GATA90G11M_14 |
| GGAA30H04_14 |
| AGAT131_14 |
| ATA069P_14 |
| GATA169E06_14 |
| ATAC026P_14 |
| GATA91G06_14 |
| GATA51F04P_14 |
| GATA045_14 |
| ATGG002_14 |
| ATT198Z_14 |
| GATA143C02_15 |
| GAAA1C11_15 |
| TAT032Z_15 |
| GATA153F11_15 |
| AATA053_15 |
| GATA63B12P_15 |
| TTAT027P_15 |
| AGAT073P_15 |
| GATA22F01_15 |
| TAGA031Z_15 |
| TTTA028_16 |
| ATA67B07P_16 |
| GATA5H07M_16 |
| GATA86C08P_16 |
| ATA10H10P_16 |
| AAT226_16 |
| TTAT023Z_16 |
| CATA002Z_16 |
| GATA143D05_16 |
| GATA151C03P_16 |
| ATA103C03P_16 |
| AAT107_16 |
| GATA140E03_16 |
| TCTA026_16 |
| MFD466-TTA001_16 |
| GATG013M_16 |
| ATACC001_16 |
| ATA063_16 |
| CTAT003_17 |
| GATA158H04_17 |
| AGAT132_17 |
| GATA64B04P_17 |
| GATA10H07P_17 |
| AAT083_17 |
| ATA58E08ZP_17 |
| GATA169F02_17 |
| GGAA19G04_17 |
| AAT245_17 |
| GATA31B11_17 |
| GATA63G01_17 |
| TTCA006M_17 |
| AAT095_17 |
| CTG008_18 |
| ATCT050_18 |
| AGAT060_18 |
| GATA036_18 |
| AGAT127_18 |
| GATA062_18 |
| GATA85D10_18 |
| GATA183H03Z_18 |
| GATA173A03_18 |
| GATA73D05_18 |
| ATAG089P_18 |
| GATA129F05_18 |
| GATA157H01_18 |
| AGAT138P_18 |
| GATA146H09_19 |
| TGA012P_19 |
| GGAA21A04_19 |
| GATA156F11_19 |
| AAT260M_19 |
| AAT257_19 |
| TTTA075P_19 |
| AAT249_19 |
| AAAT007_20 |
| AAT247_20 |
| AGAT136M_20 |
| GATA72E11_20 |
| ATTC013_20 |
| GATA81E09_20 |
| AGAT139P_20 |
| GATA142C02M_20 |
| GATA65E01_20 |
| GATA90E02_20 |
| ATCT035_20 |
| AAT269_20 |
| TATT031_20 |
| TTTA093P_20 |
| GATA163G03_21 |
| TATC057_21 |
| GATA148F04P_21 |
| AGAT057_21 |
| AGAT120_22 |
| TTA015P_22 |
| ATTT019M_22 |
| AGAT055Z_22 |
| GATA6F05P_22 |
| TCAT006ZP_22 |
| GTAT005Z_22 |
| TTAT020P_22 |
| SCA10_22 |
| GATA030P_22 |
| TCTA015M_22 |

Supporting Table 4. Heterozygosities for the different chromosomes in African and Non-African *Drosophila melanogaster* populations.

|  | X | 2nd | 3rd | 4th |
| --- | --- | --- | --- | --- |
| Bol | 0.279638 | 0.424188 | 0.473364 | 0.398943 |
| CBr | 0.366149 | 0.48481 | 0.479736 | 0.379253 |
| Bel | 0.342913 | 0.539332 | 0.498514 | 0.45126 |
| NJ | 0.399832 | 0.510512 | 0.488014 | 0.442749 |
| Pe | 0.386846 | 0.46958 | 0.467686 | 0.442403 |
| Ceb | 0.292465 | 0.341985 | 0.468202 | 0.324035 |
| Chi | 0.335515 | 0.43593 | 0.455748 | 0.244461 |
| Hchu | 0.39394 | 0.484824 | 0.516361 | 0.344072 |
| KL | 0.357502 | 0.421166 | 0.369342 | 0.139648 |
| Evo | 0.363452 | 0.480083 | 0.478161 | 0.430884 |
| KaBe | 0.353763 | 0.455886 | 0.469993 | 0.312338 |
| Kat | 0.376406 | 0.415154 | 0.470461 | 0.361496 |
| Neu | 0.367482 | 0.455807 | 0.464617 | 0.395093 |
| Np | 0.37897 | 0.43282 | 0.464585 | 0.3322 |
| Tex | 0.374406 | 0.45215 | 0.478854 | 0.38337 |
| TBO | 0.334377 | 0.399629 | 0.415102 | 0.434324 |
| Cyg | 0.324585 | 0.423752 | 0.420501 | 0.437785 |
| Woo | 0.405625 | 0.490139 | 0.506064 | 0.456264 |
| Mor | 0.381392 | 0.487267 | 0.488903 | 0.441557 |
| ZS | 0.673664 | 0.634763 | 0.615621 | 0.472304 |
| ZW | 0.674757 | 0.671523 | 0.678399 | 0.440401 |

Supporting Table 5. Results of the linear models.

| Regions’ Feature | R^2^ | *p value* |
| --- | --- | --- |
| Number of genes | 0.03 | 0.52 |
| Region length | 0.02 | 0.662 |
| Number of transposable elements | 0.01 | 0.69 |
| Number of non-coding RNA | 0.02 | 0.65 |
| Presence/Absence of Inversion | 0.08 | 0.29 |
| Expected Heterozygosity excluding the African populations | 0.09 | 0.24 |
| Expected Heterozygosity including the African populations | 0.11 | 0.2 |
| Average *F_ST_* | 0.14 | 0.16 |
| Expected Theta excluding the African populations | 0.12 | 0.186 |
| Expected Theta including the African populations | 0.24 | 0.06 |
| Recombination Rate | 0.04 | 0.48 |

Supporting Table 6. Relationship between the number of loci and divergence for the estimate of population structure.

|  | Loci randomly drawn | | | | | | | | | |
| --- | --- | --- | --- | --- | --- | --- | --- | --- | --- | --- |
|  | 10 | 20 | 30 | 40 | 50 | 60 | 70 | 80 | 90 | 100 |
| Number of times the expected clustering solution (5 clusters) occurred among 1000 replicates | | | | | | | | | | |
| *F_ST_* 0.01 | 0 | 0 | 0 | 0 | 0 | 0 | 0 | 0 | 0 | 0 |
| *F_ST_* 0.05 | 233 | 467 | 669 | 799 | 887 | 921 | 968 | 985 | 991 | 990 |
| *F_ST_* 0.1 | 941 | 995 | 1000 | 1000 | 1000 | 1000 | 1000 | 1000 | 1000 | 1000 |
| *F_ST_* 0.15 | 997 | 1000 | 1000 | 1000 | 1000 | 1000 | 1000 | 1000 | 1000 | 1000 |
| Number of different clustering solutions among the 1000 replicates | | | | | | | | | | |
| *F_ST_* 0.01 | 3 | 1 | 1 | 1 | 1 | 1 | 1 | 1 | 1 | 1 |
| *F_ST_* 0.05 | 50 | 38 | 33 | 19 | 12 | 13 | 11 | 8 | 7 | 5 |
| *F_ST_* 0.1 | 11 | 5 | 1 | 1 | 1 | 1 | 1 | 1 | 1 | 1 |
| *F_ST_* 0.15 | 4 | 1 | 1 | 1 | 1 | 1 | 1 | 1 | 1 | 1 |
| Number of clustering solutions different than 5 clusters and with a posterior probability higher than 0.95. | | | | | | | | | | |
| *F_ST_* 0.01 | 998/998 | 1000/1000 | 1000/1000 | 1000/1000 | 1000/1000 | 1000/1000 | 1000/1000 | 1000/1000 | 1000/1000 | 1000/1000 |
| *F_ST_* 0.05 | 603/836 | 478/945 | 308/977 | 193/992 | 112/999 | 78/999 | 32/1000 | 15/1000 | 9/1000 | 10/1000 |
| *F_ST_* 0.1 | 59/1000 | 5/1000 | 0/1000 | 0/1000 | 0/1000 | 0/1000 | 0/1000 | 0/1000 | 0/1000 | 0/1000 |
| *F_ST_* 0.15 | 2/999 | 0/1000 | 0/1000 | 0/1000 | 0/1000 | 0/1000 | 0/1000 | 0/1000 | 0/1000 | 0/1000 |

Supporting Figure 1. Microsatelite marker design. Schematic of a chromosome where one region wherein microsatellites where genotyped has been magnified. The enlarged region shows eight marks in red which represent the microsatellite positions within the region. The X, 2^nd^ and 3^rd^ chromosomes have 5 such regions along their length and the 4^th^ chromosome due to its small size only one.


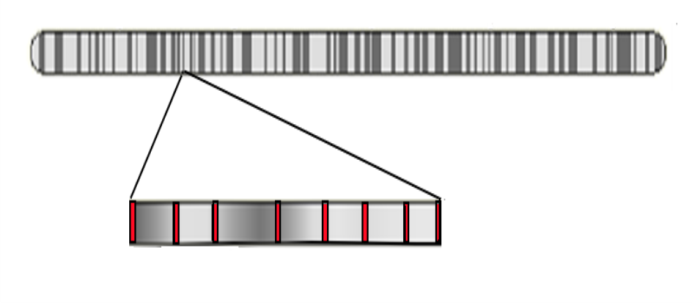


Supporting Figure 2. Clustering solutions for each chromosome.

X Chromosome


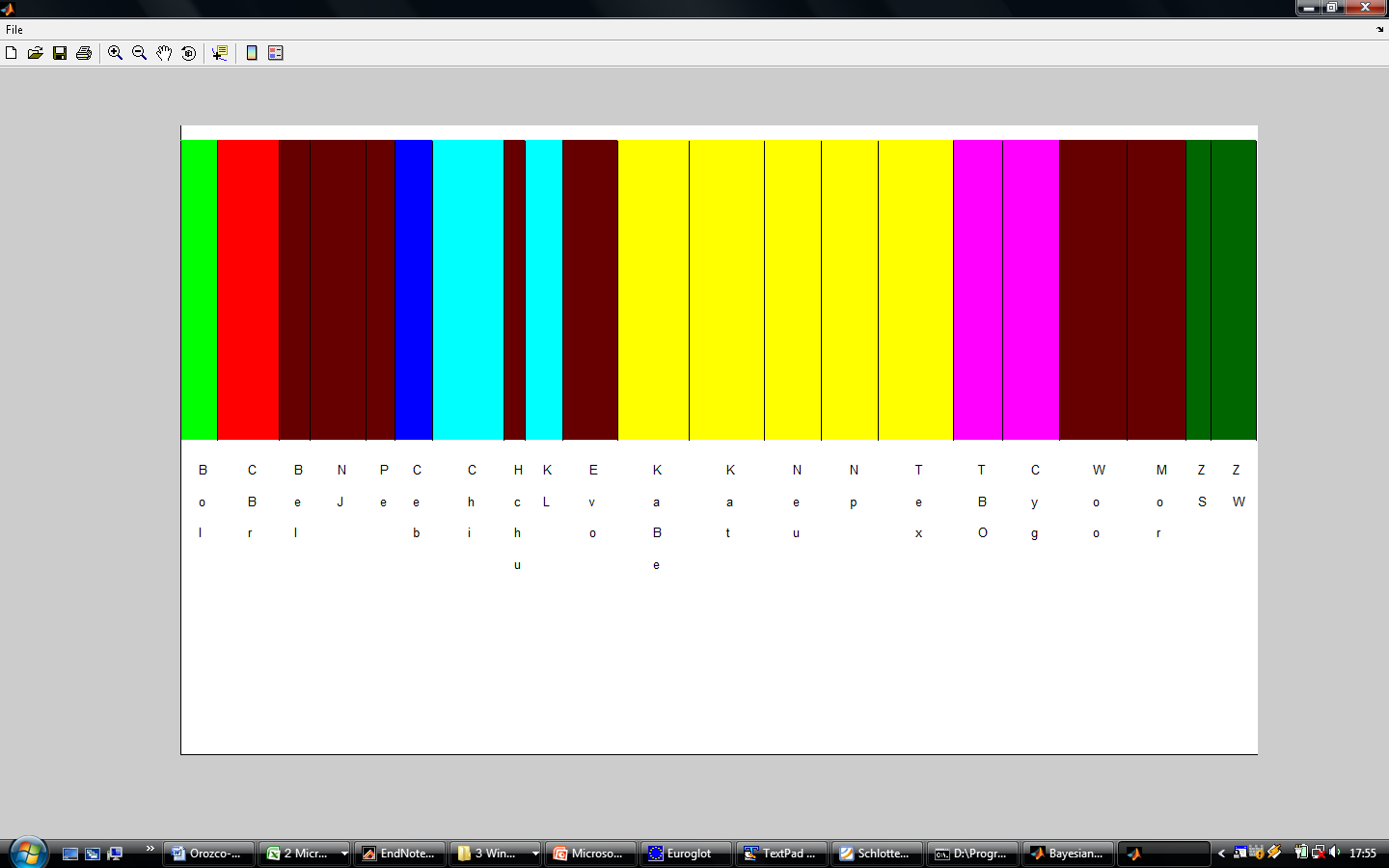


2^nd^ Chromosome


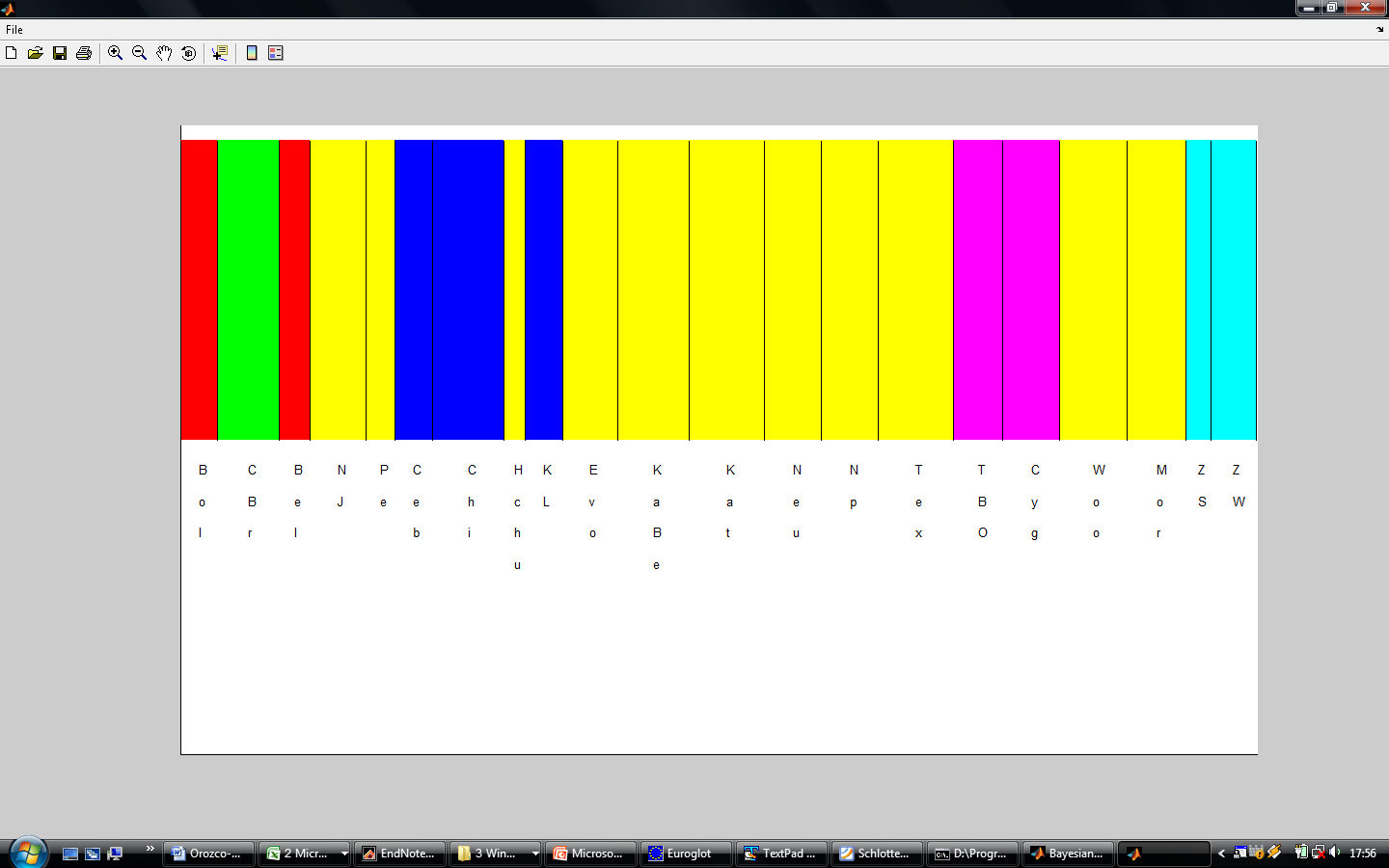


3^rd^ Chromosome


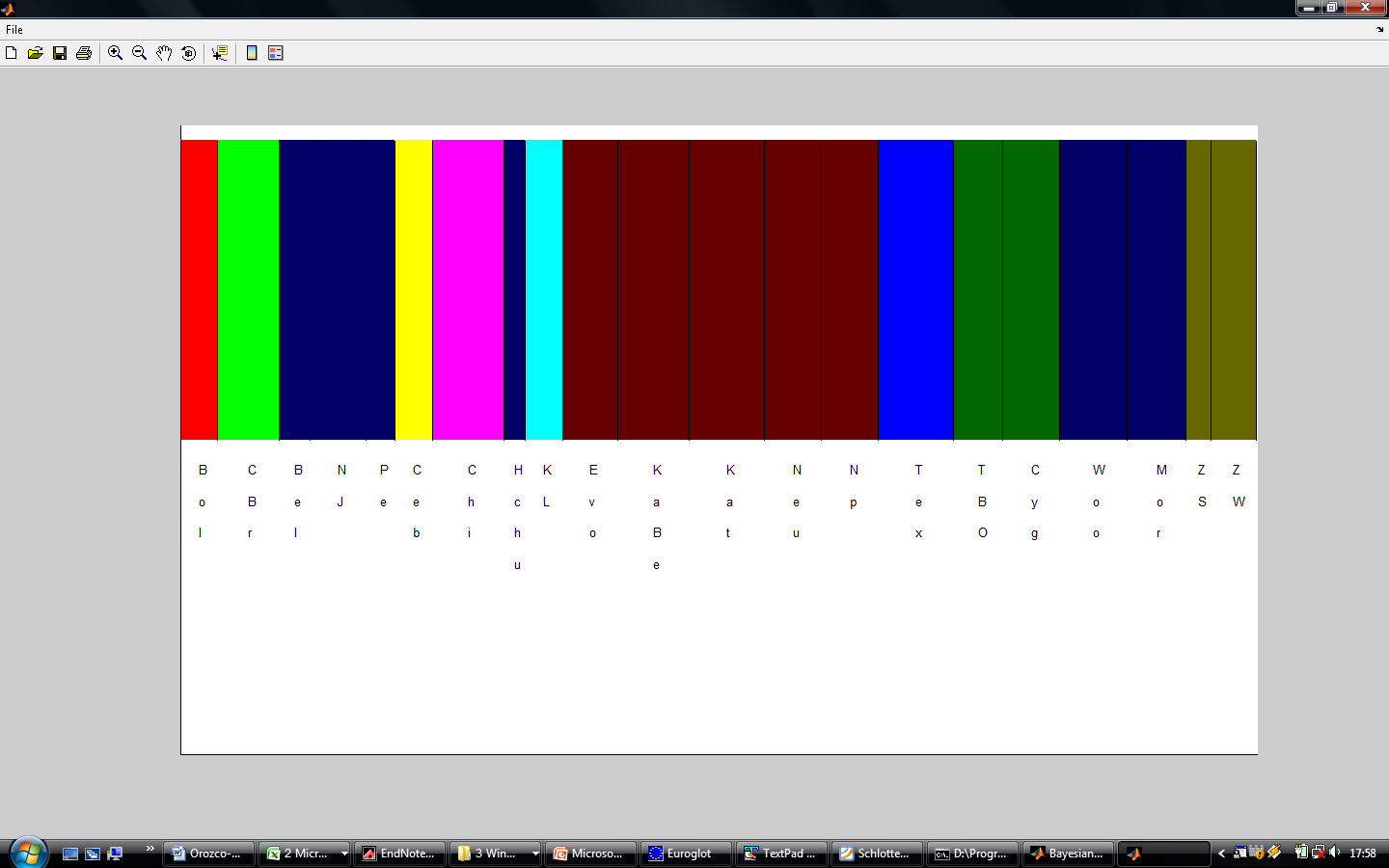


4^th^ Chromosome


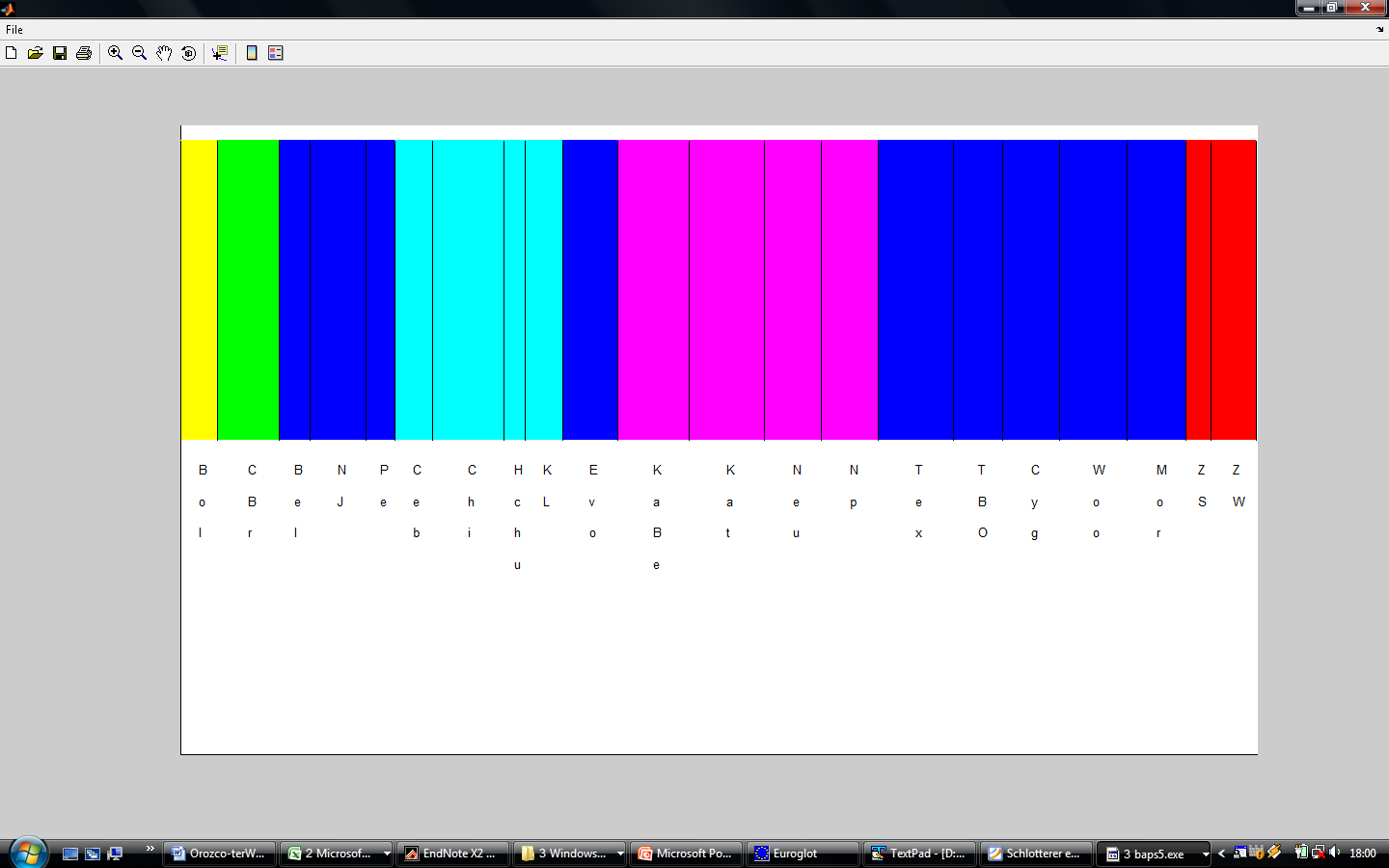


Supporting Figure 3. Clustering solutions for each genomic region. Each clustering solution is labeled with its correspondent region label.

Xr1


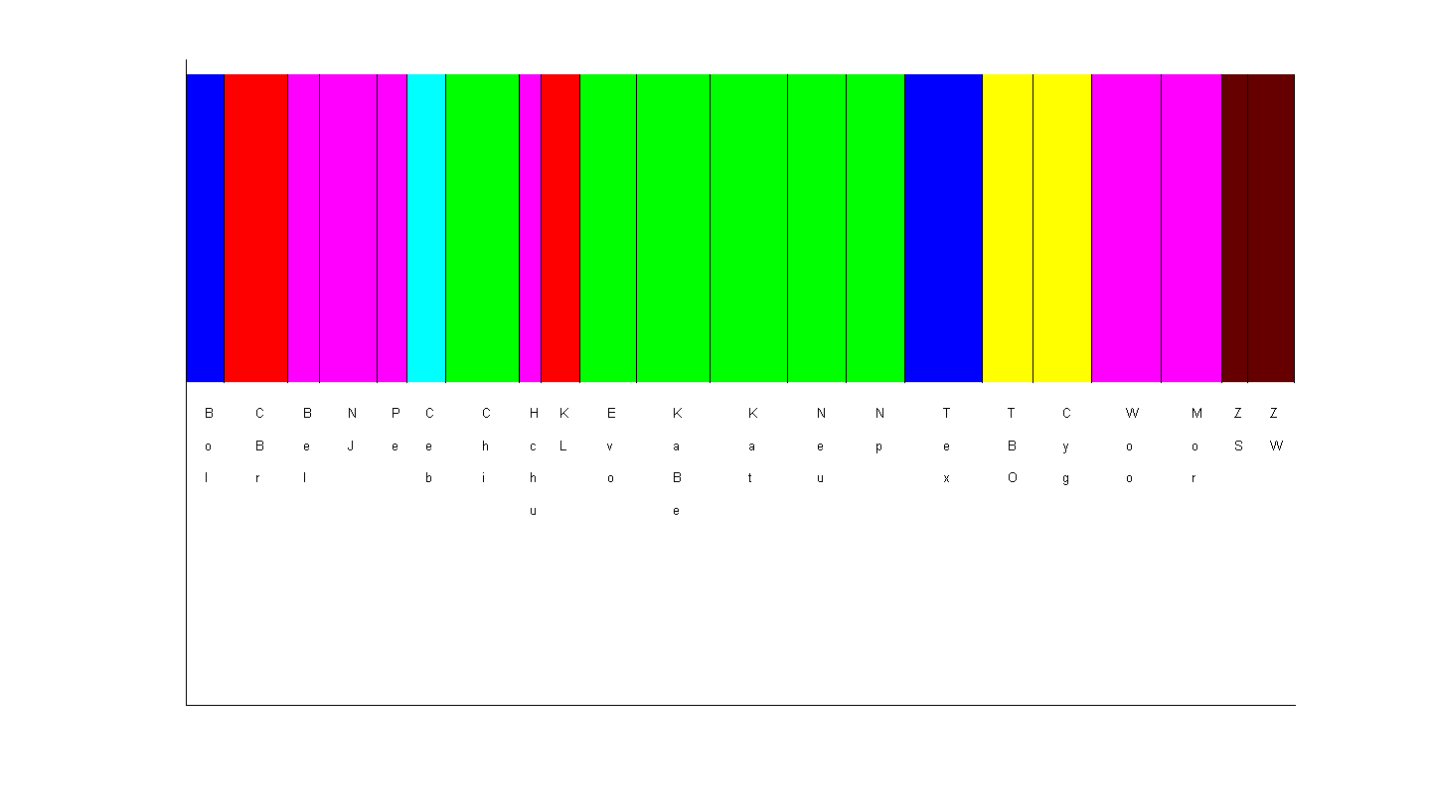


Xr2


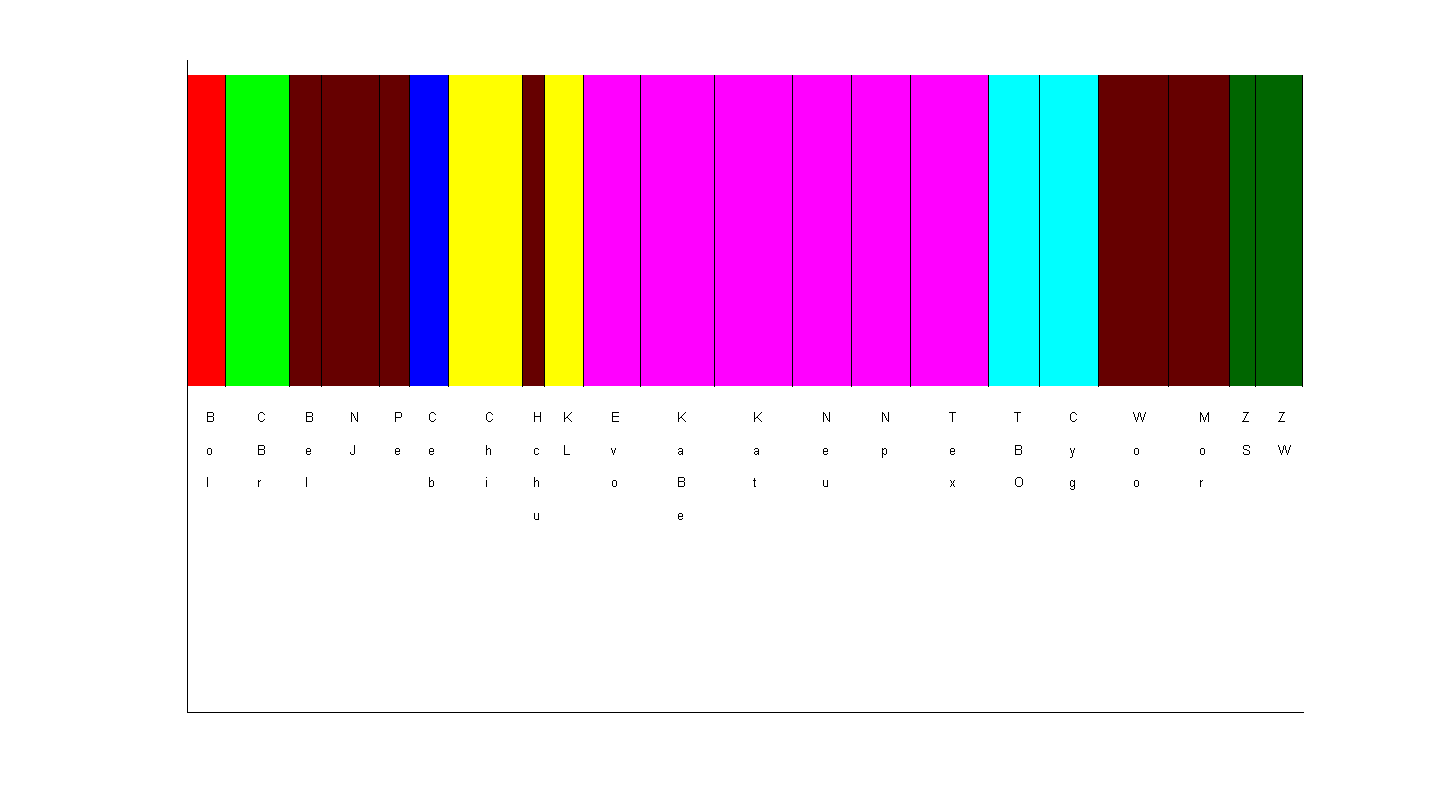


Xr3 m8 (8 microsatellites)


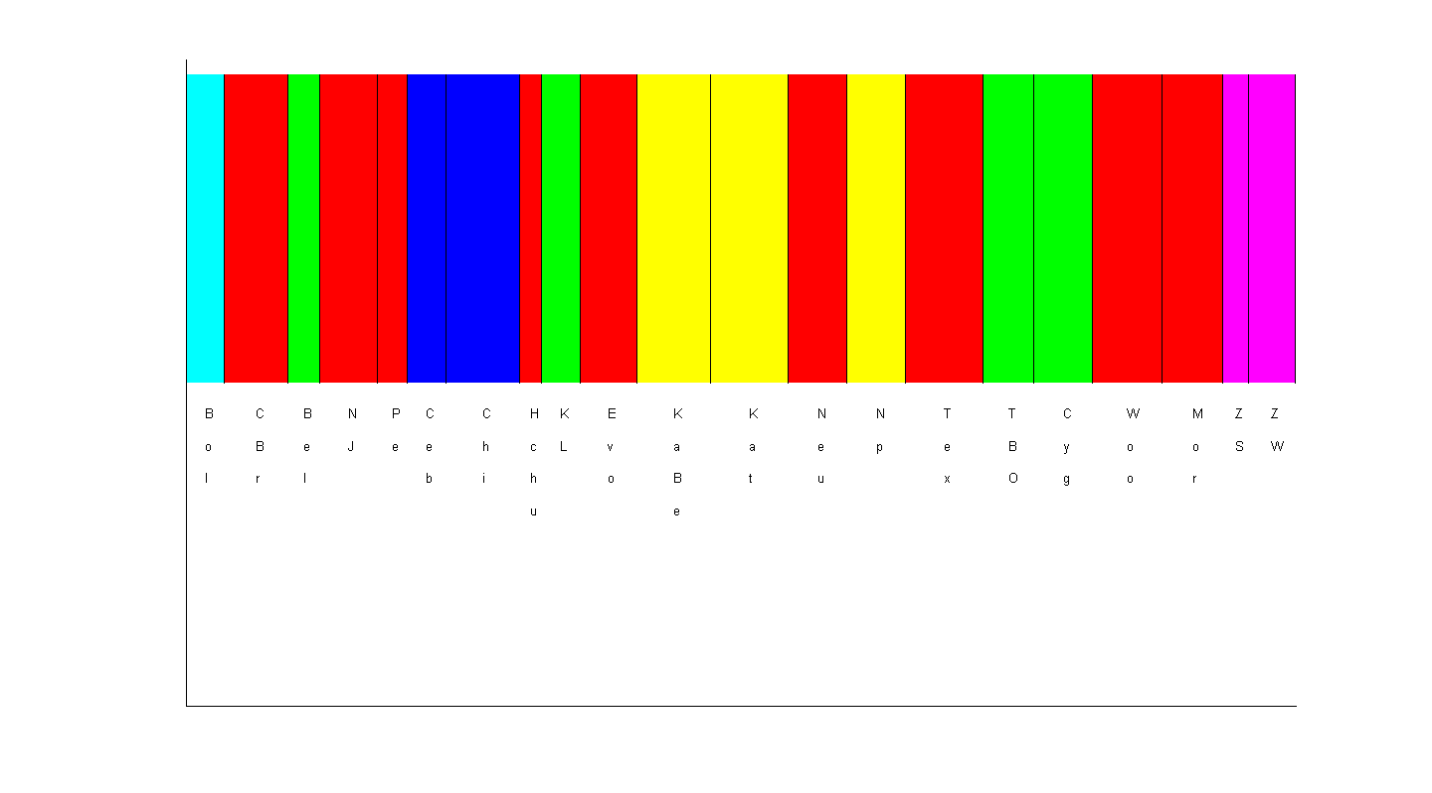


Xr3 m12 (12 microsatellites)


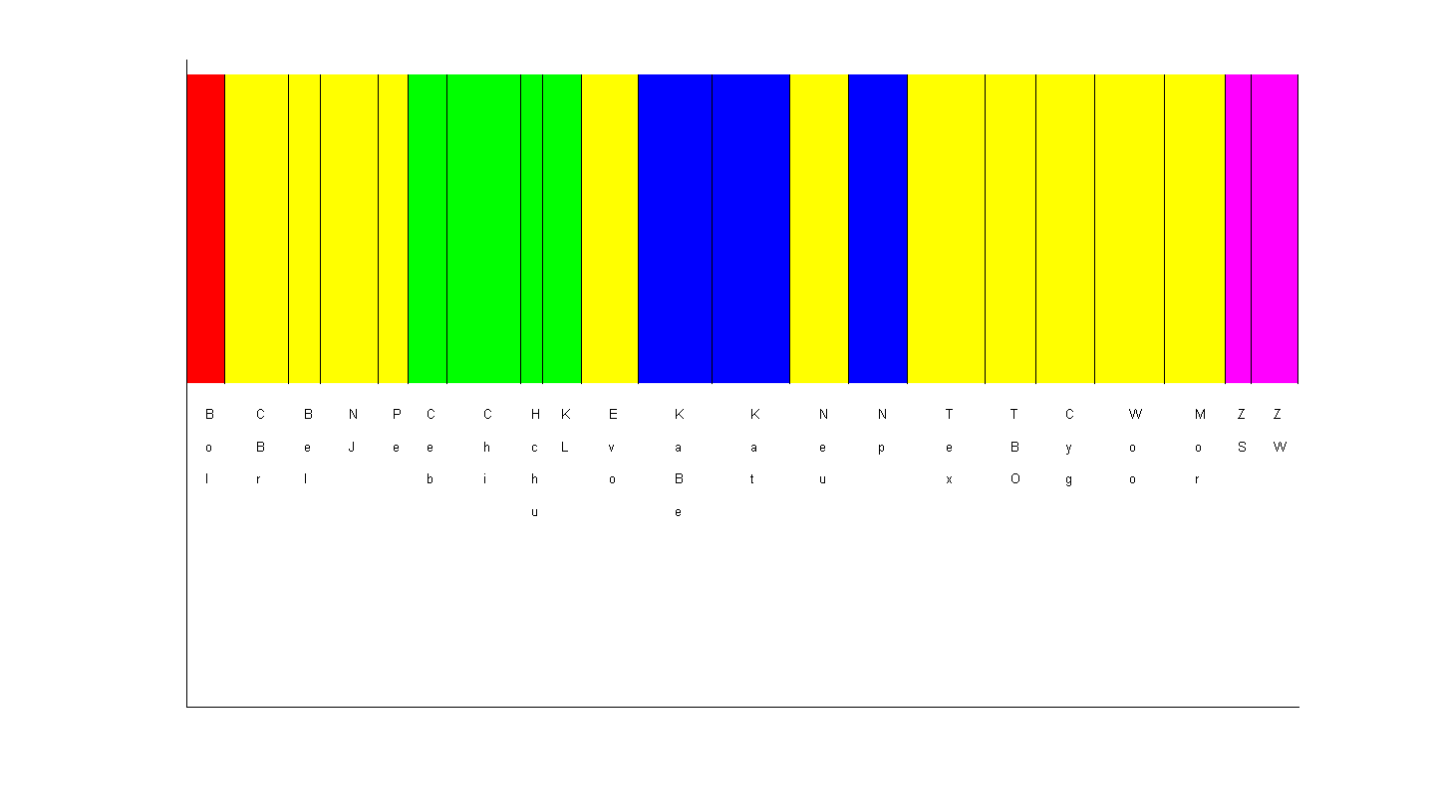


Xr4 m8 (8 microsatellites)


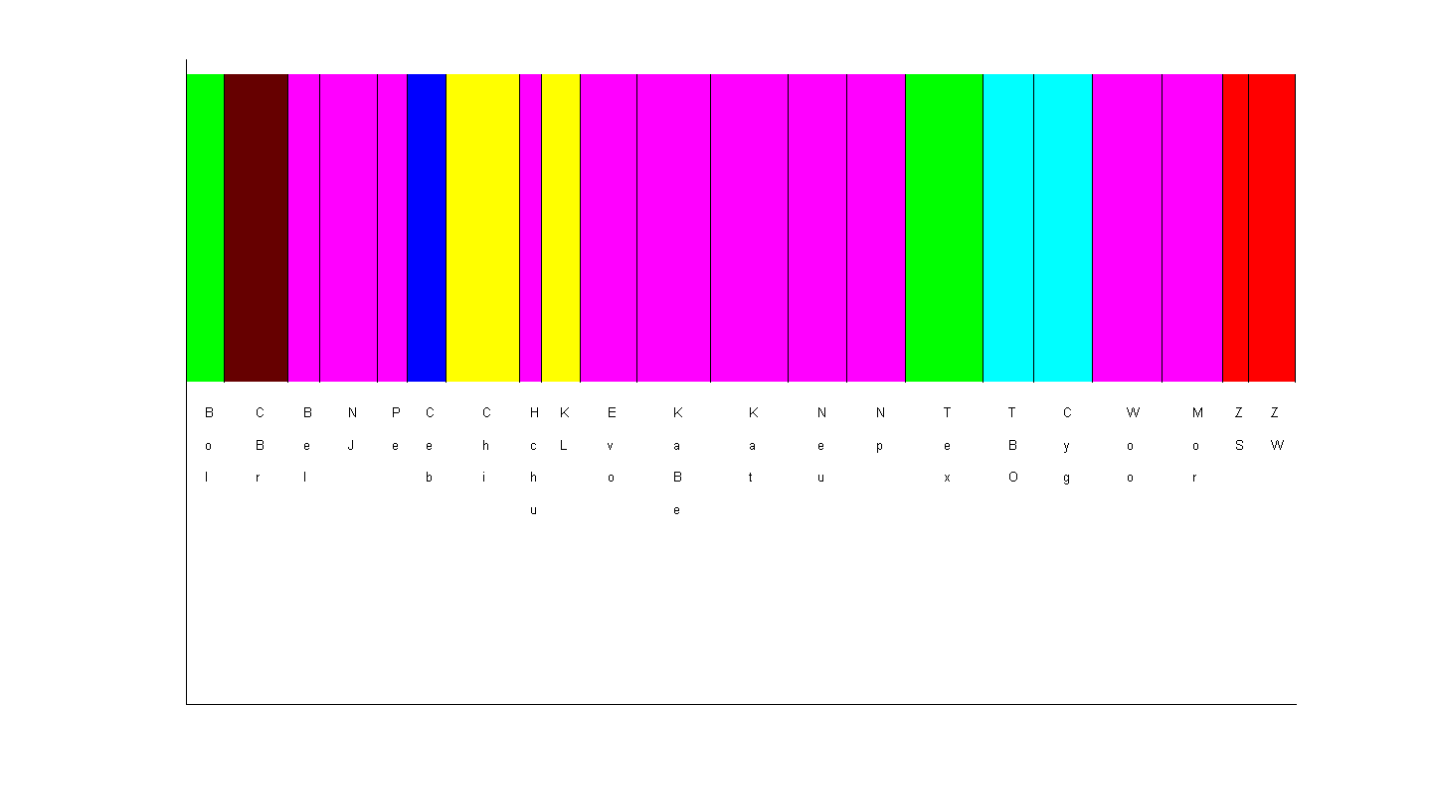


Xr4 m 12 (12 microsatellites)


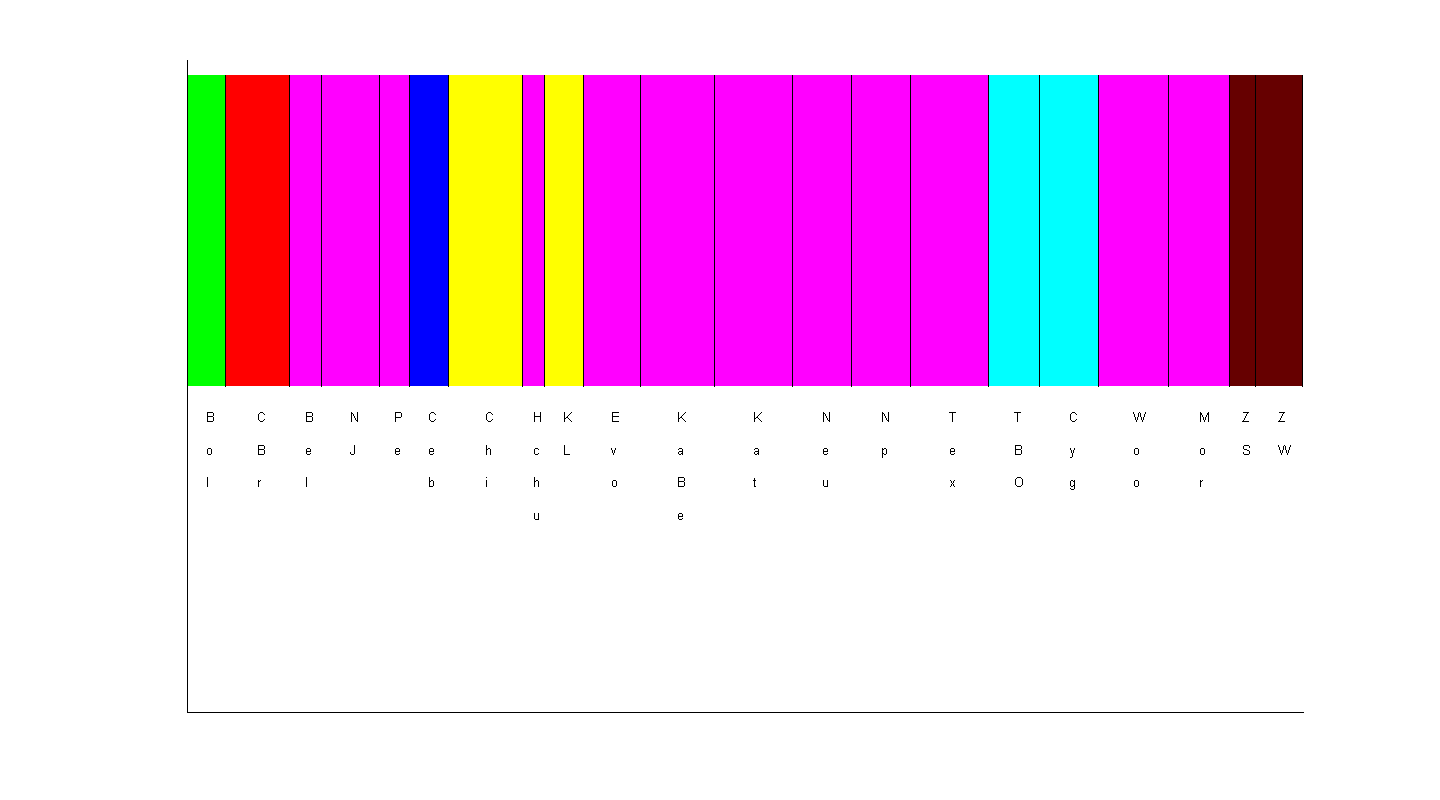


Xr5,


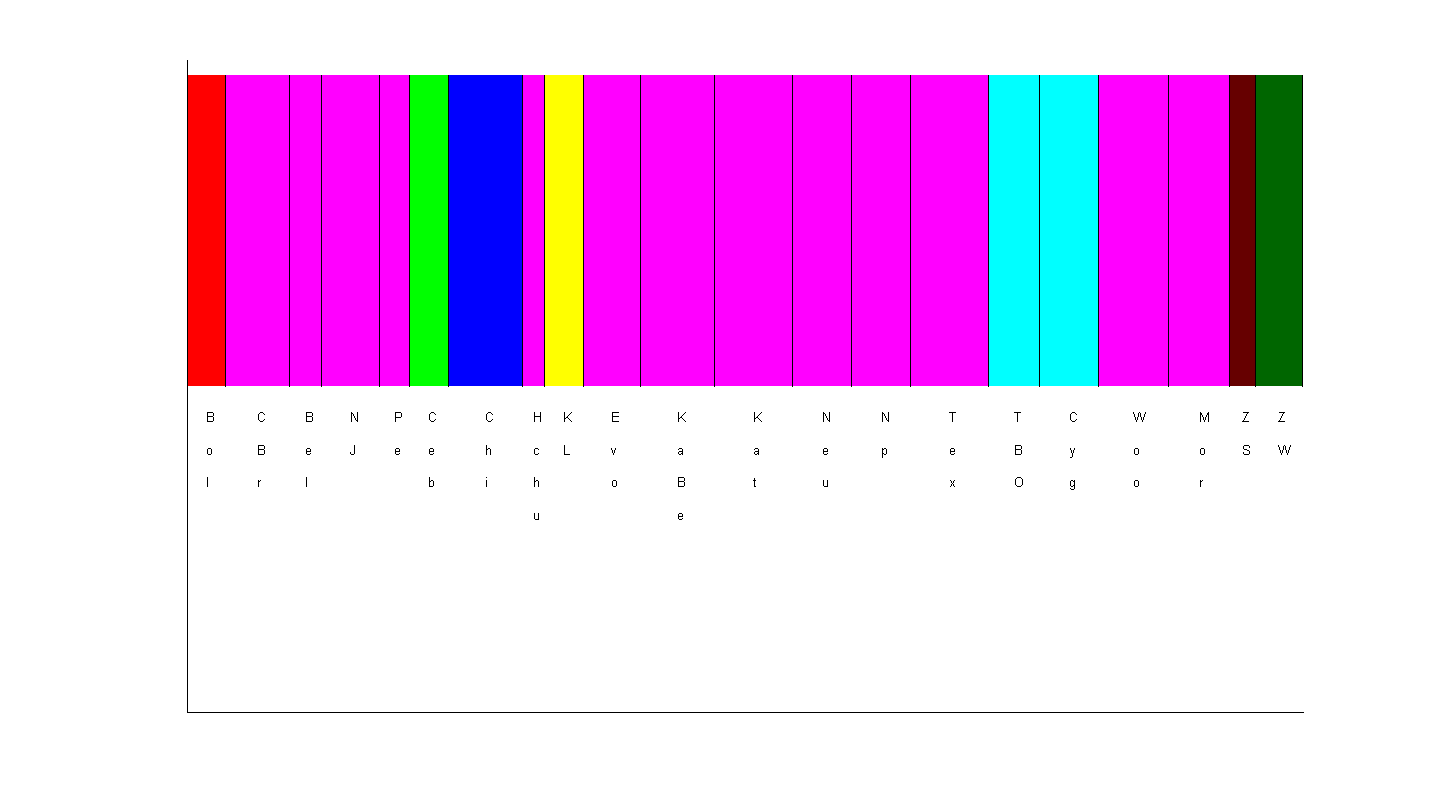


2r1


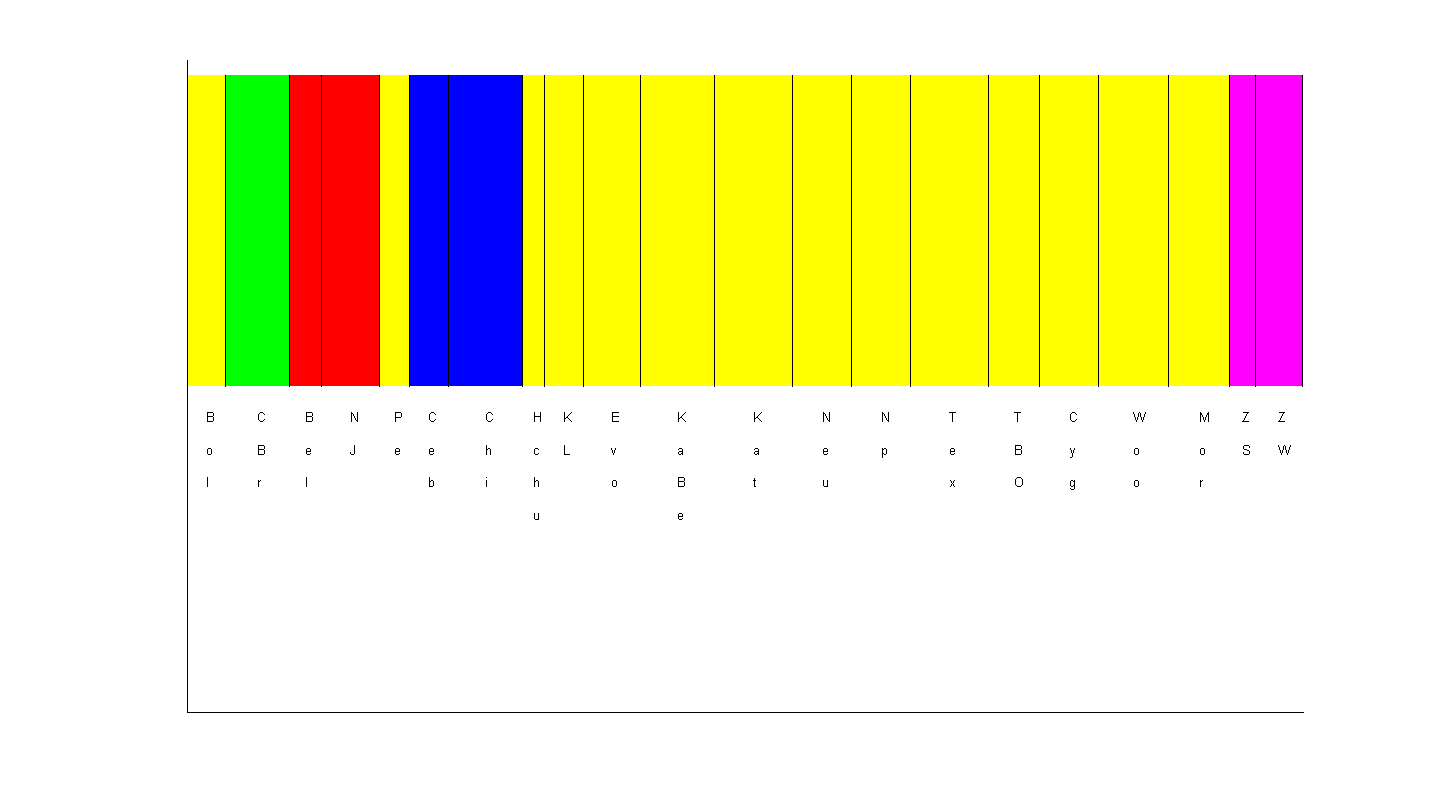


2r2


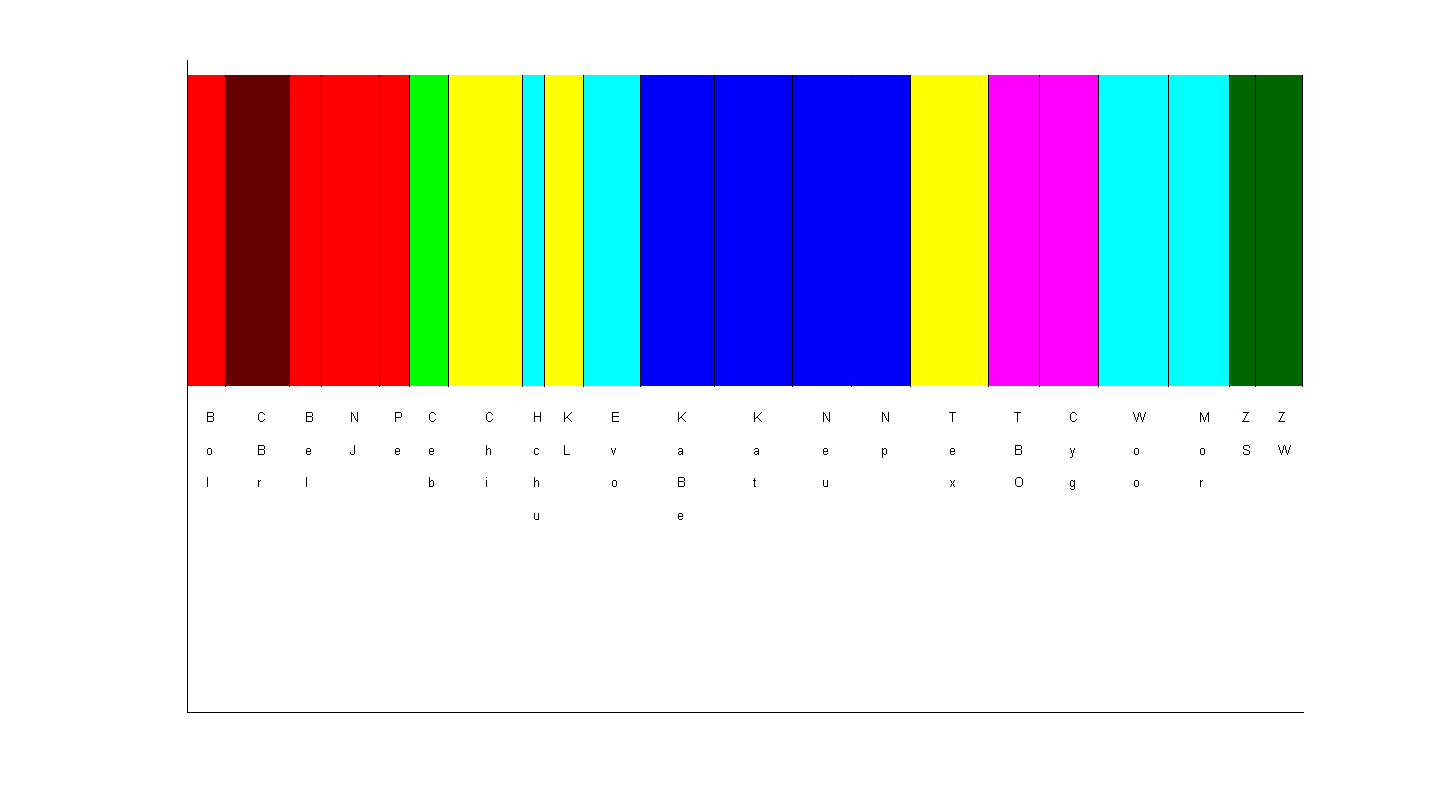


2r3


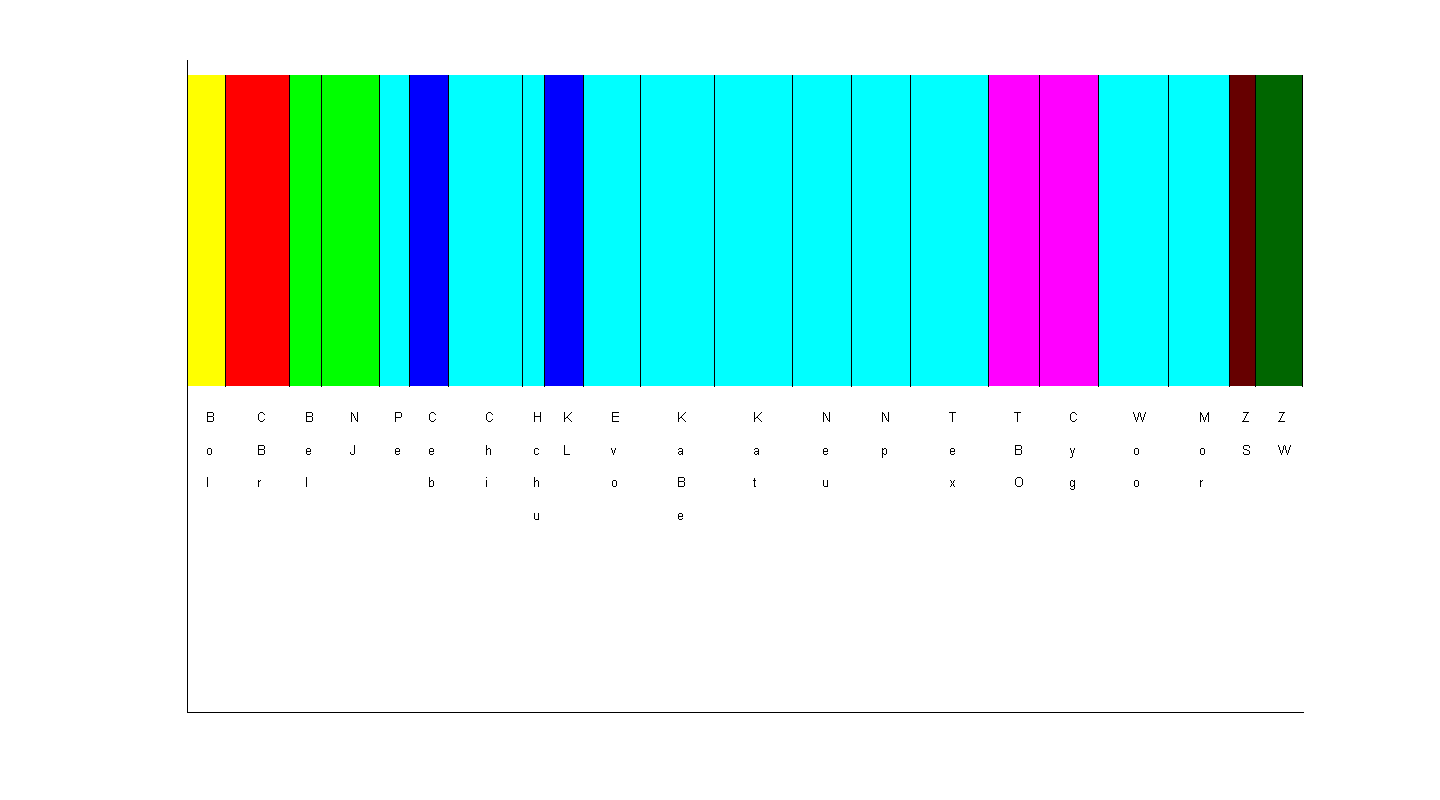


2r4


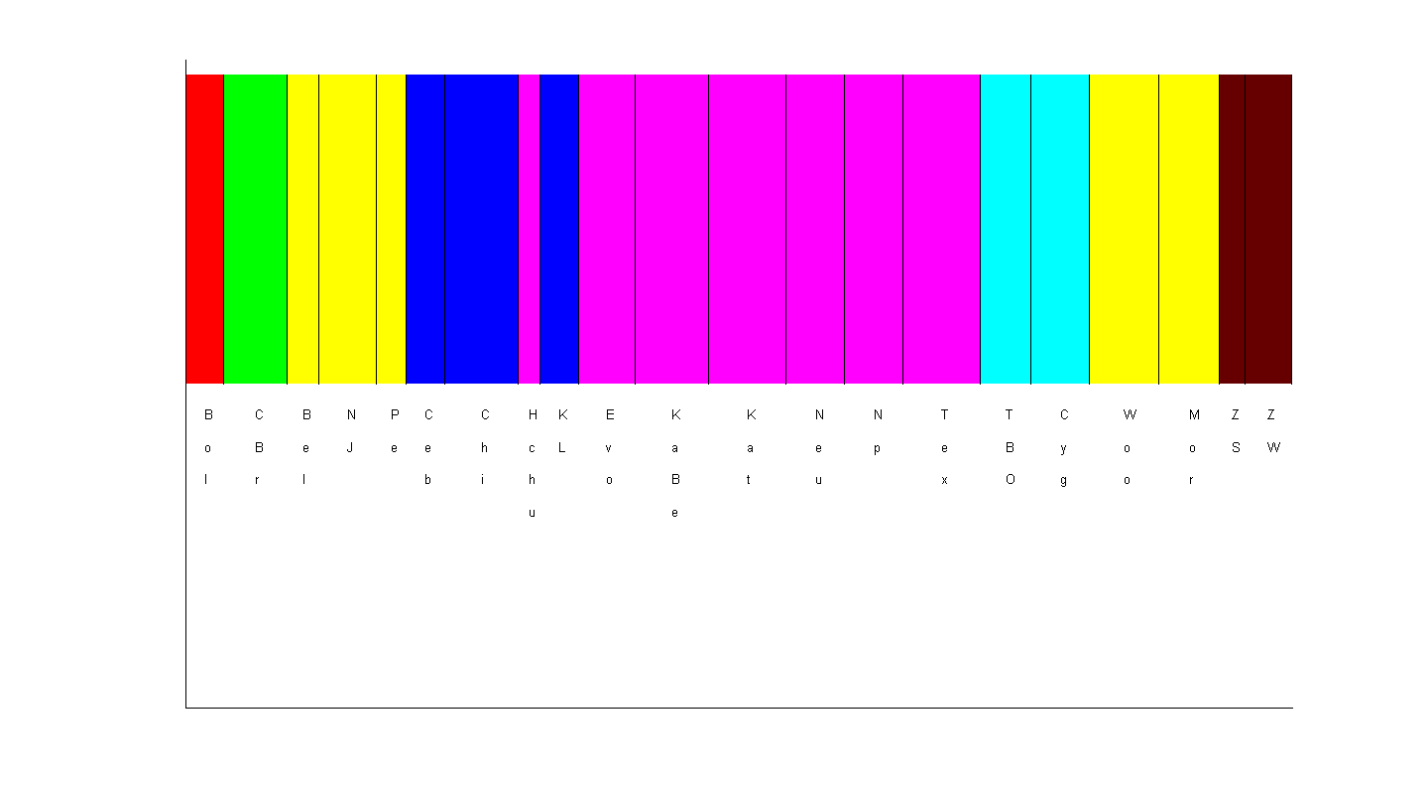


2r5


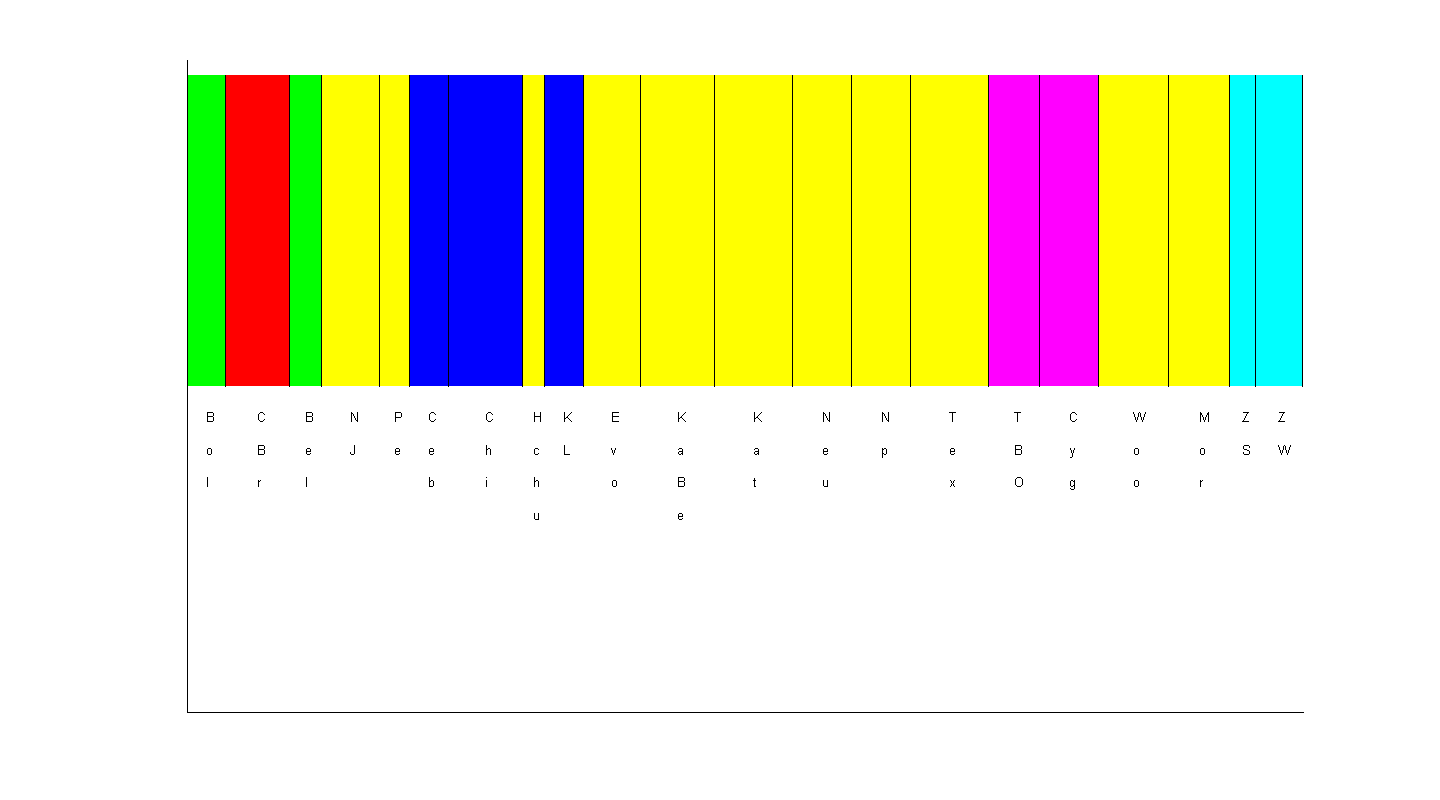


3r1


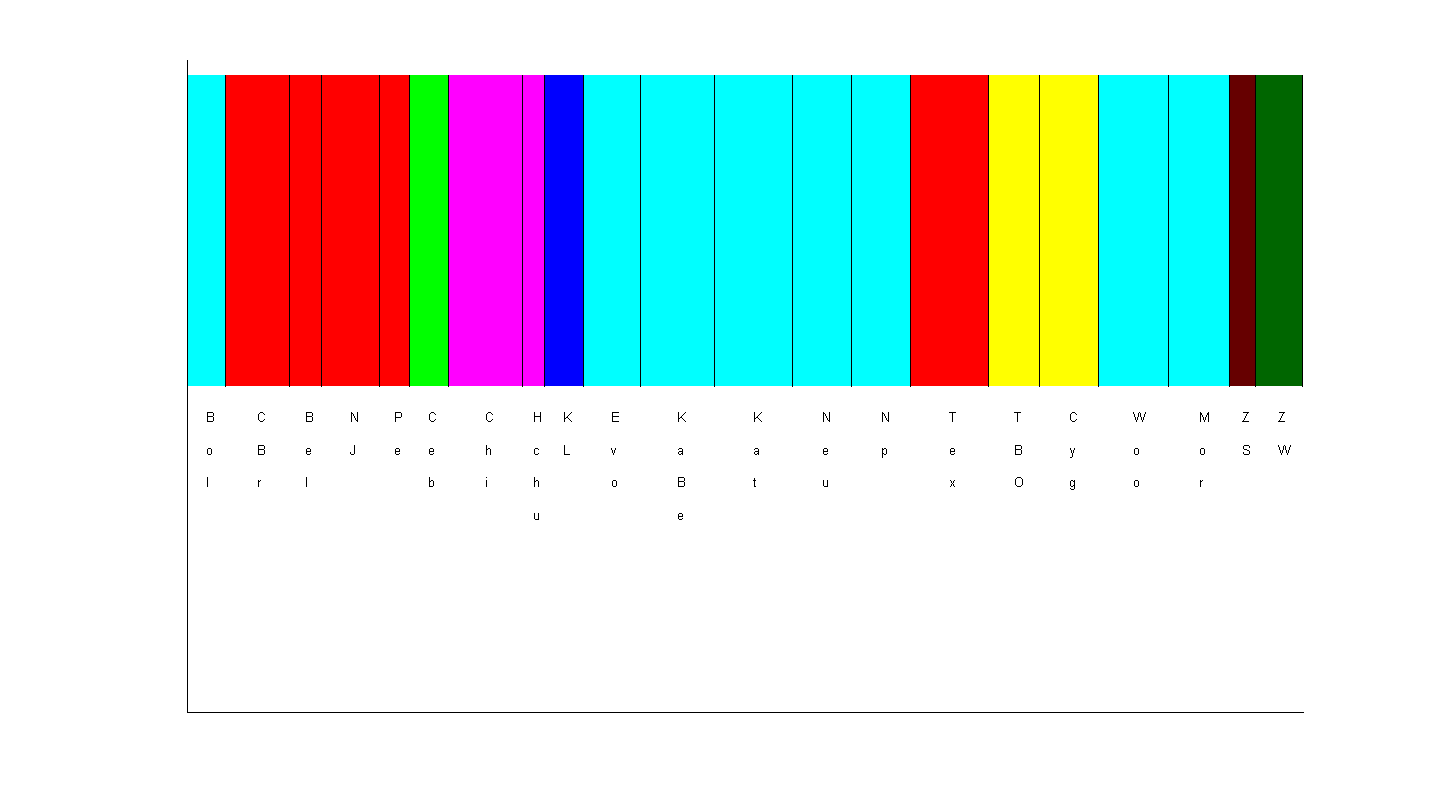


3r2


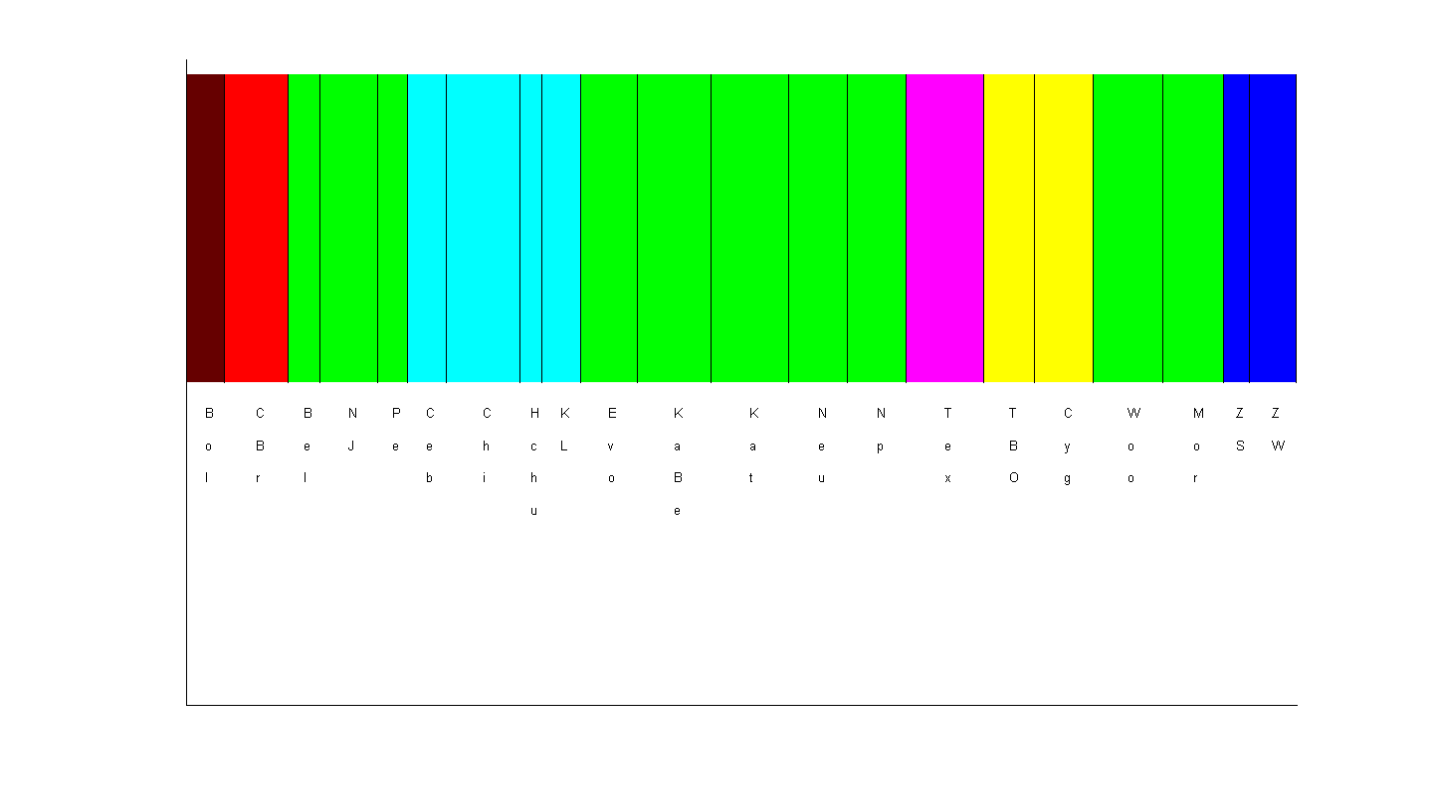


3r3


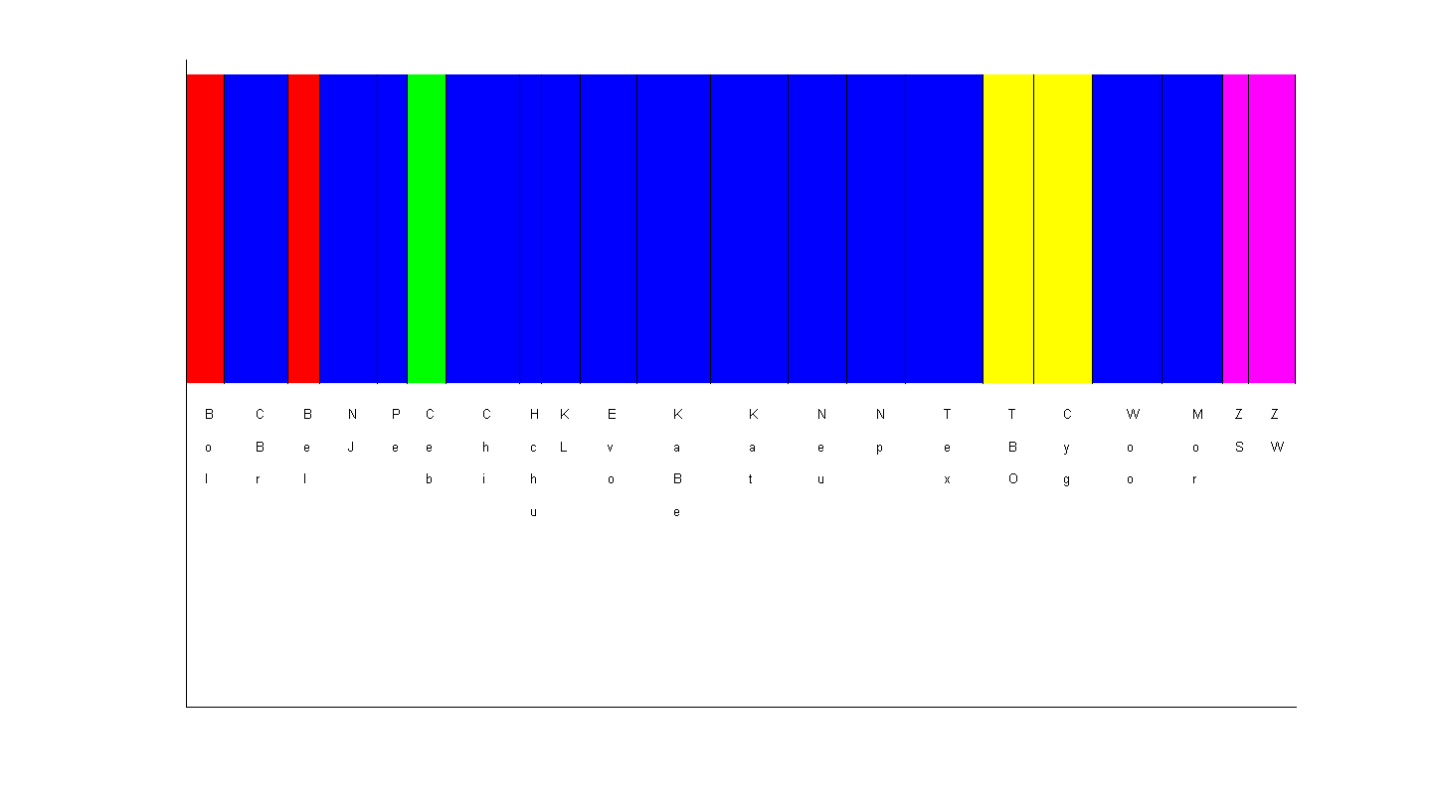


3r4


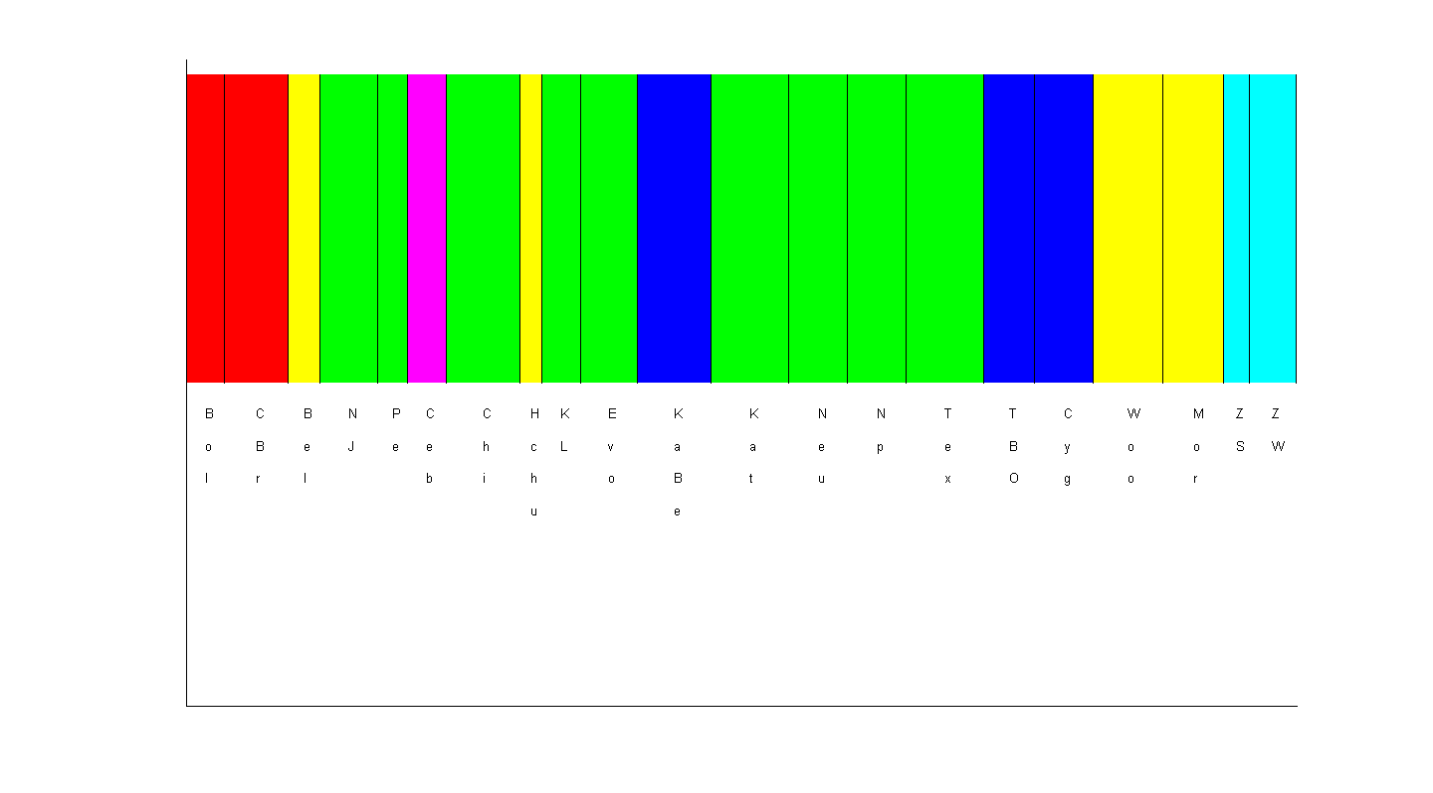


3r5


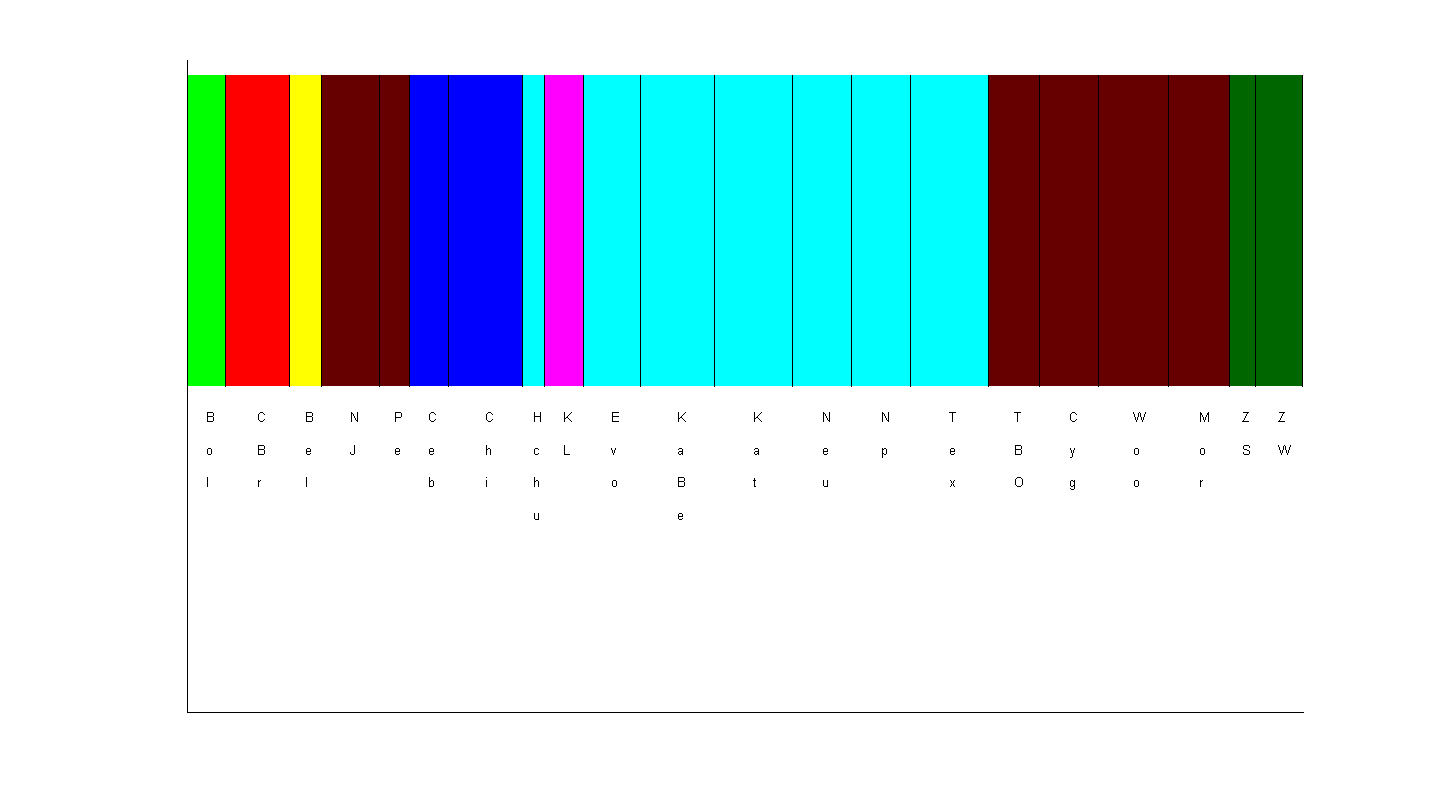


4


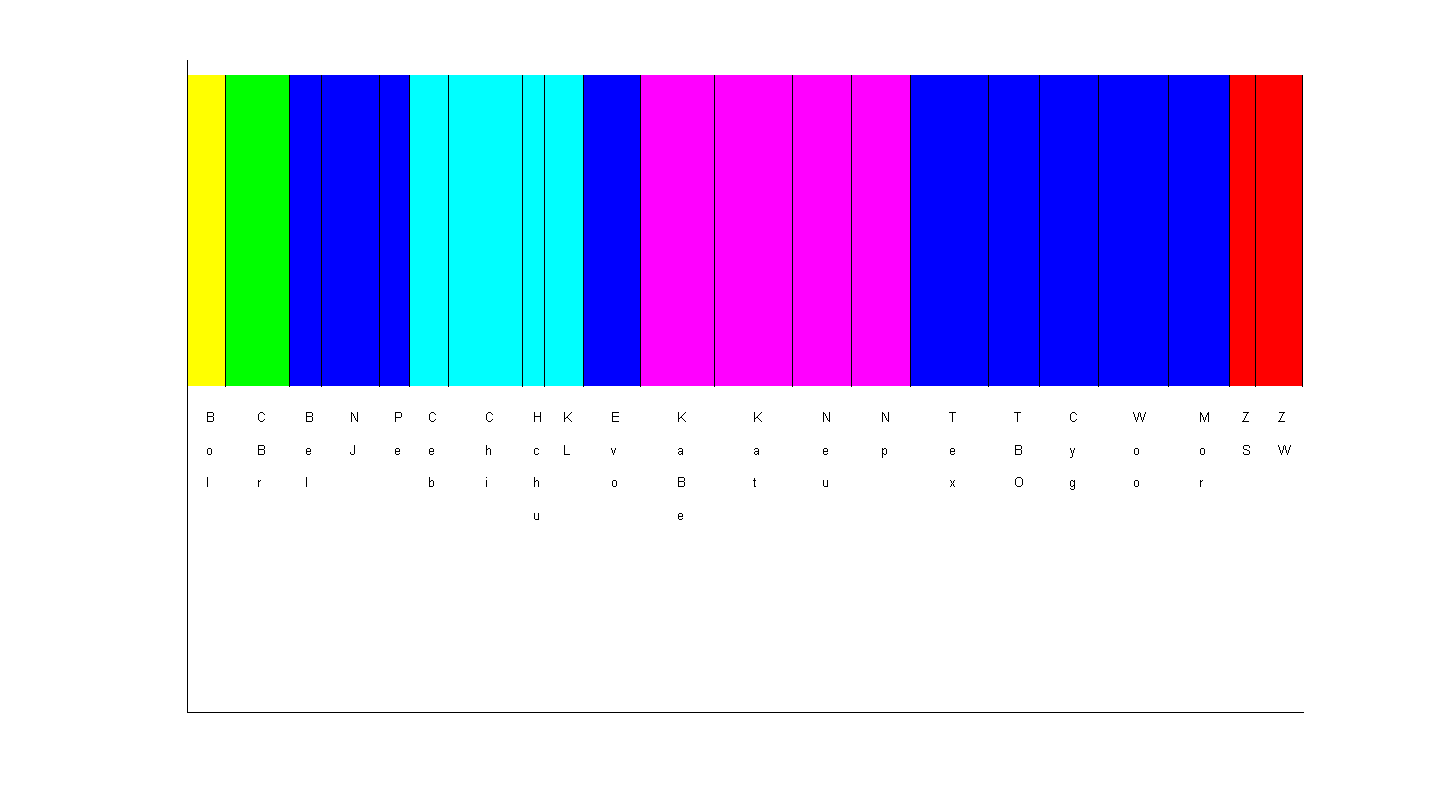


Supporting Figure 4. Histograms of the ml-differences distribution for the regions against each of the chromosomes. A) comparison for the X regions, B) comparison for the 2^nd^ chromosome regions, C) comparison for the 3^rd^ chromosome regions and D) comparison for the region in the 4^th^ chromosome.

A)

B)

C)

D)

Supporting Figure 5. Correlation plots of the region’s properties against the region’s ranking position. Log(ml): logartithm of the marginal likelihood of the regions’ clustering solutions.

A) Log(ml) versus number of genes in the region.


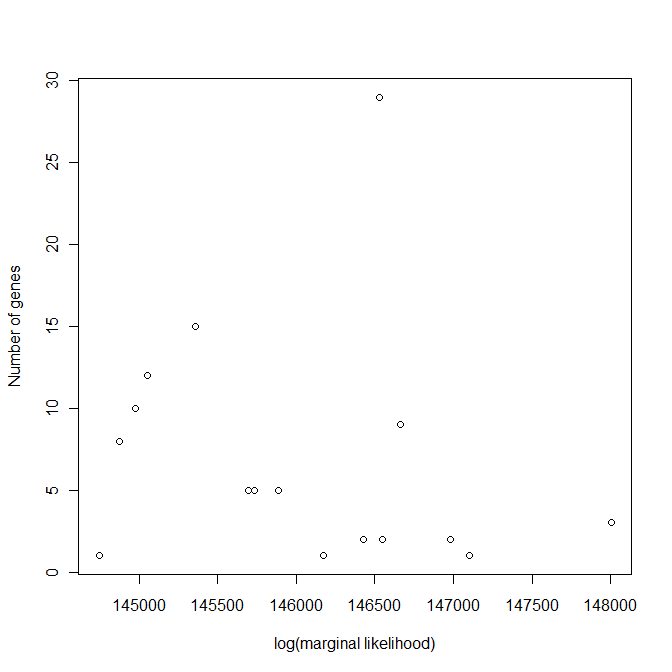


B) Log(ml) versus regions’ length.


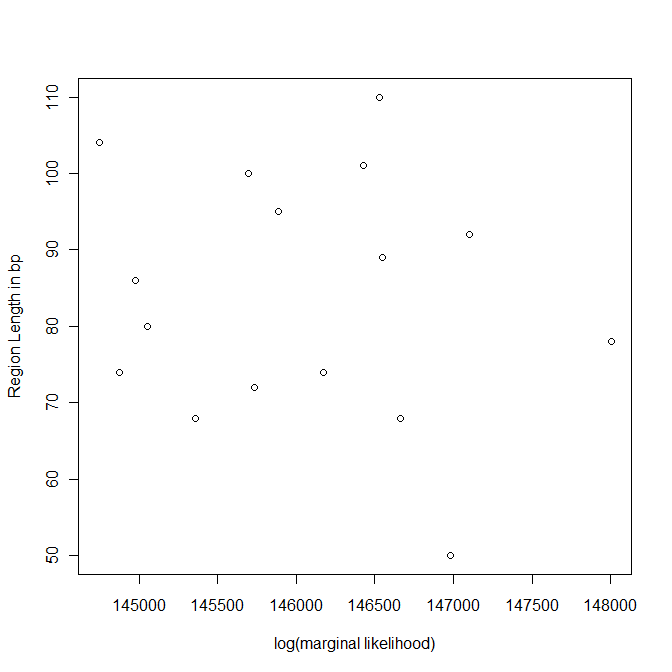


C) Log(ml) versus number of transposable elements in the region.


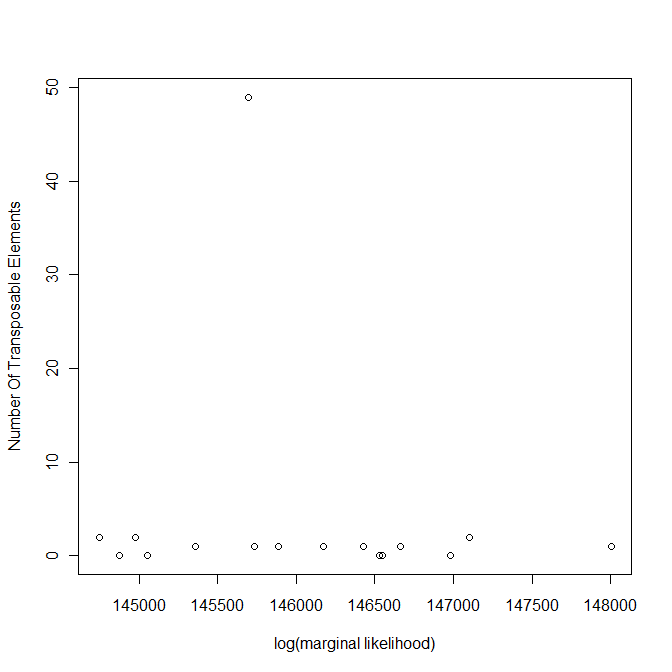


D) Log(ml) versus number of non-coding RNAs in the region.


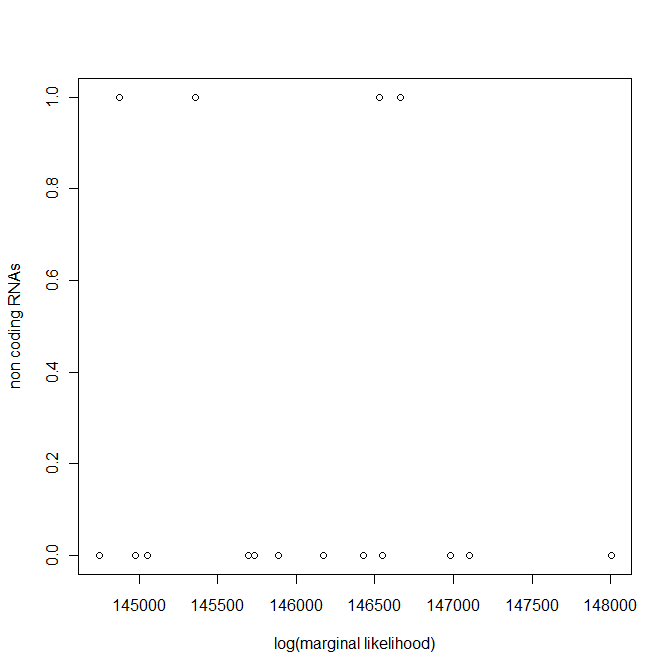


E) Log(ml) versus presence/absence of inversions in the regions.


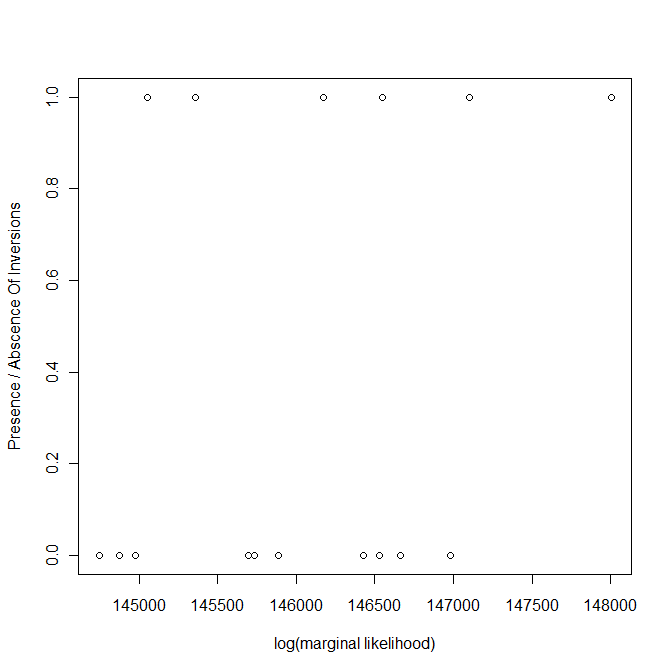


F) Log(ml) versus expected heterozygosity excluding the African populations.


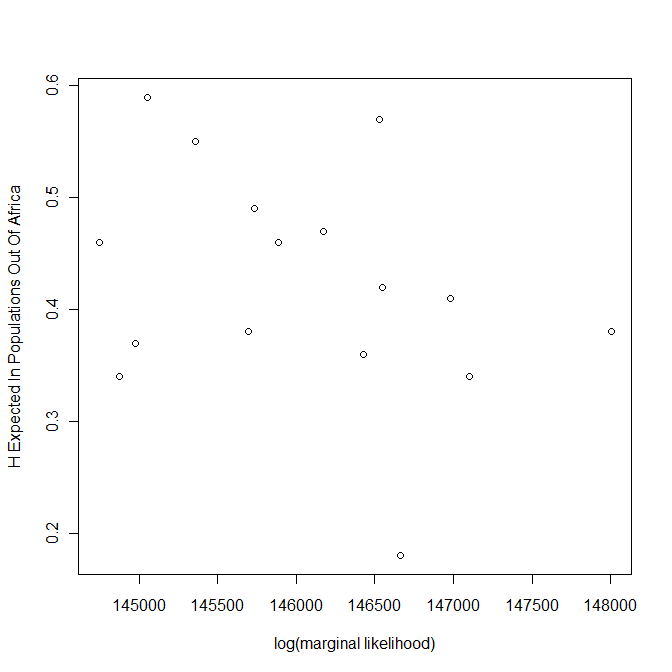


G) Log(ml) versus expected heterozygosity including the African populations.


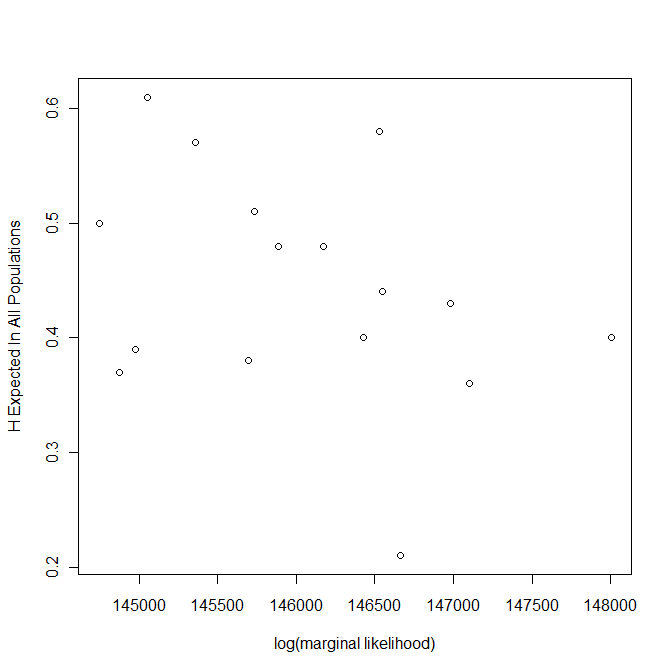


H) Log(ml) versus regions’ average *F_ST_*


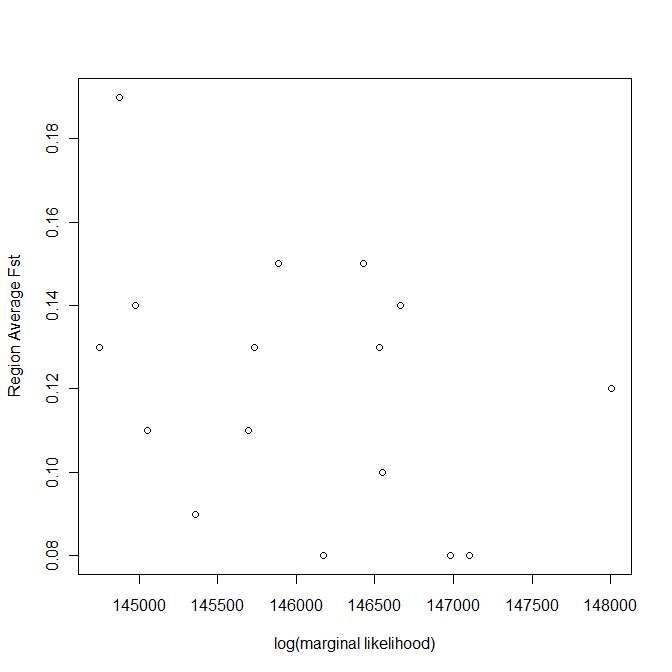


I) Log(ml) versus expected theta excluding the African populations


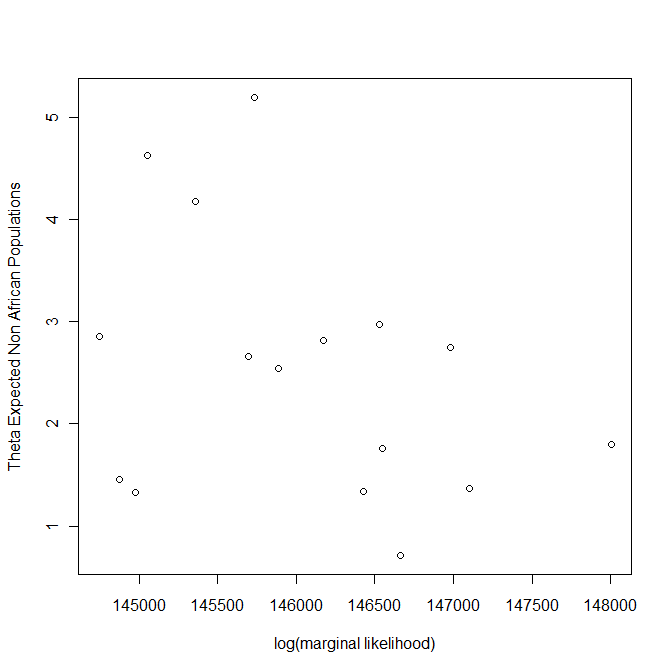


J) Log(ml) versus expected theta including the African populations


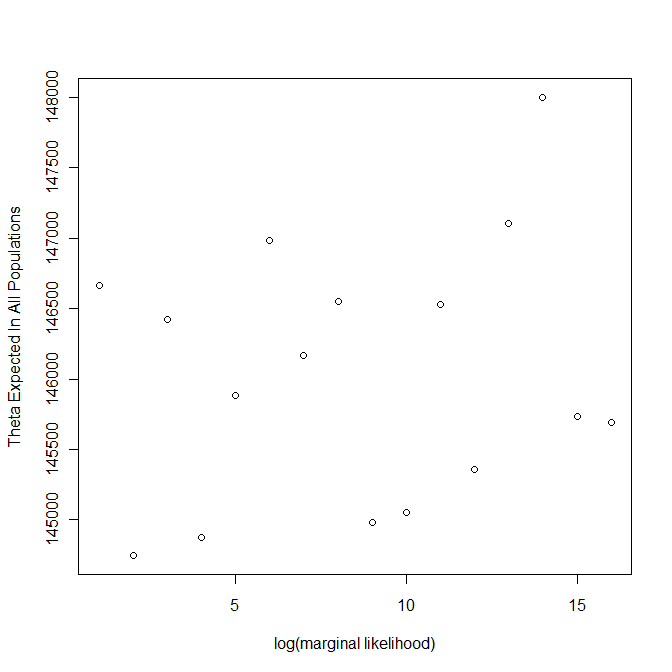


K) Log(ml) versus the regions’ recombination rate.


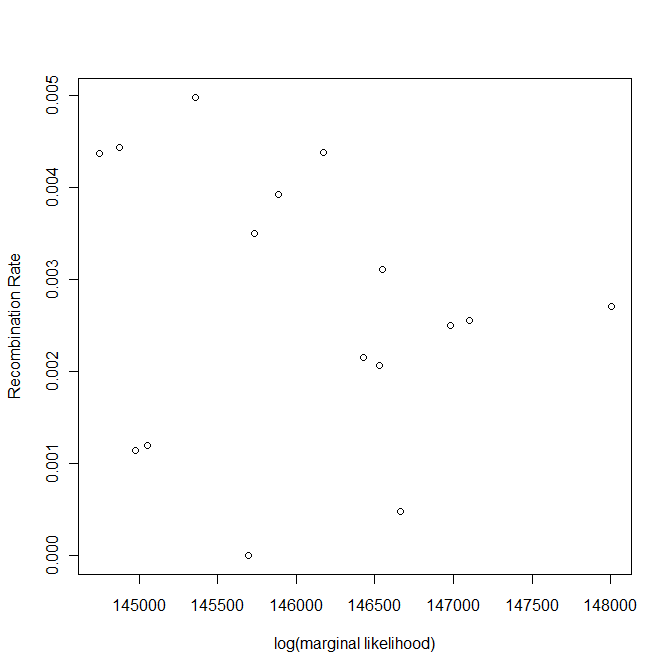


Supporting Figure 6. Simplified illustration of the effect of genealogical lineage sorting. Each line represents the frequency with which the true clustering solution (i.e. A(B,C)) occurs among 1000 random draws of a set of *n* loci (*n* from 10 to 100). The lines correspond to the results from drawing loci from a distribution of markers where the proportion of loci resulting in the true population structure is: 20% (dashed blue), 30% (dotted gray), 40% (dashed-dotted green), 50% (long-dashed orange), 60% (dashed red). When the loci that result in the true population structure occur with a frequency of 70% or higher in the genome 10 or more loci result in the expected clustering solution (solid-black line).


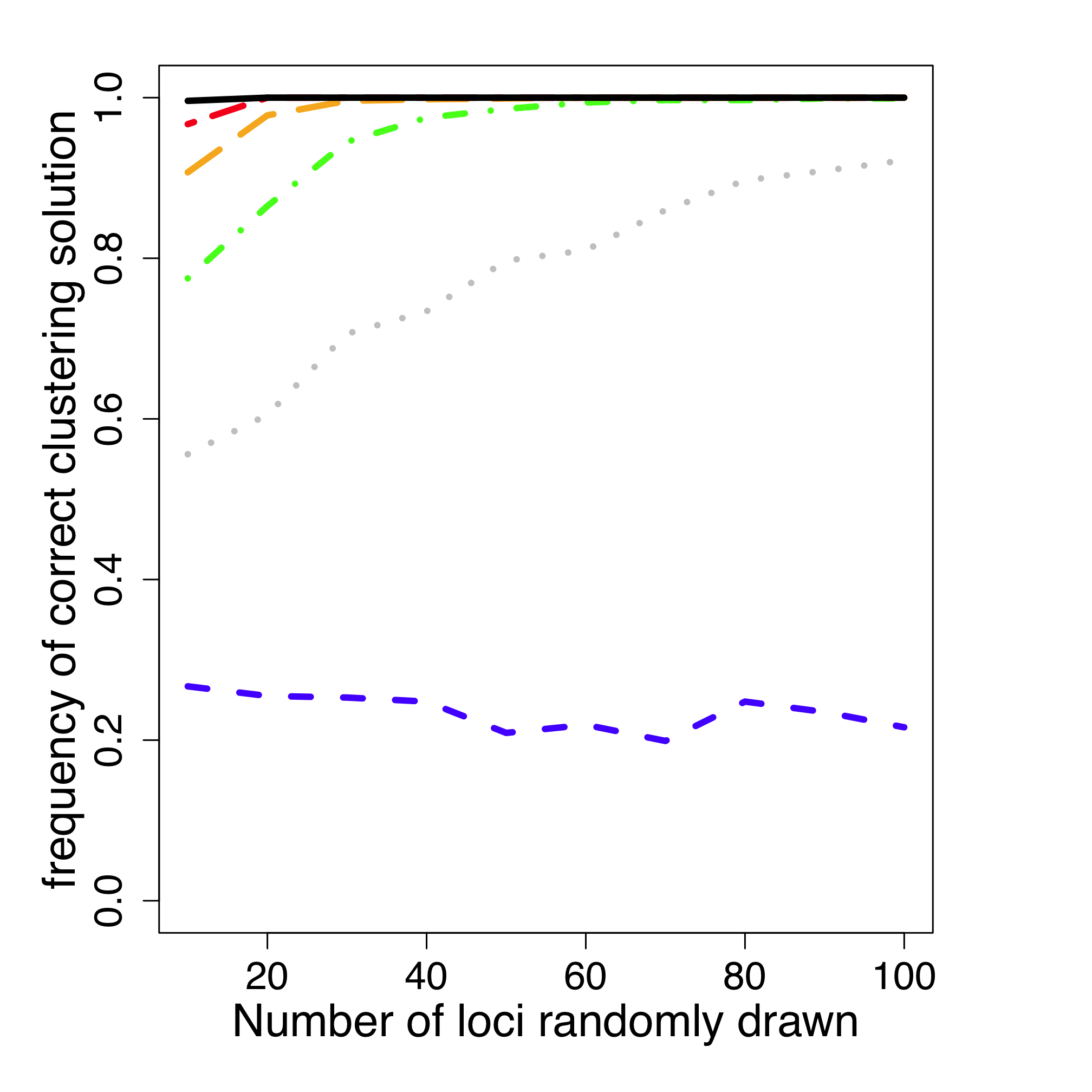


Supporting Figure 7. Relationship between population differentiation and genealogical inference. We used computer simulations to determine the frequency of correctly inferred clustering solutions in relationship to the number of loci used and population differentiation when using two Bayesian methods to infer population structure, i.e. BAPS and Structure. Results are shown for 50 simulations of 5 populations with average *F*_ST_ values of: 0.01 (dashed grey line), 0.05 (solid blue line) and 0.1 (dashed light blue line). For a detailed explanation of the simulated datasets see Materials & Methods.
